# Embedding a self-supporting MOF-based molecular sieve membrane into an electrolyzer for boosting electroreduction of CO_2_ in air and flue gas to HCOOH

**DOI:** 10.1093/nsr/nwaf329

**Published:** 2025-08-12

**Authors:** Da-Shuai Huang, Yu Wang, Yi Tang, Jia-Run Huang, Pei-Xian Li, Cheng-Peng Liang, Zhen-Hua Zhao, Pei-Qin Liao, Xiao-Ming Chen

**Affiliations:** MOE Key Laboratory of Bioinorganic and Synthetic Chemistry, GBRCE for Functional Molecular Engineering, School of Chemistry, IGCME, Sun Yat-sen University, Guangzhou 510275, China; Key Laboratory of Special Functional and Smart Polymer Materials of Ministry of Industry and Information Technology, Xi'an Key Laboratory of Functional Organic Porous Materials, School of Chemistry and Chemical Engineering, Northwestern Polytechnical University, Xi'an 710072, China; MOE Key Laboratory of Bioinorganic and Synthetic Chemistry, GBRCE for Functional Molecular Engineering, School of Chemistry, IGCME, Sun Yat-sen University, Guangzhou 510275, China; MOE Key Laboratory of Bioinorganic and Synthetic Chemistry, GBRCE for Functional Molecular Engineering, School of Chemistry, IGCME, Sun Yat-sen University, Guangzhou 510275, China; MOE Key Laboratory of Bioinorganic and Synthetic Chemistry, GBRCE for Functional Molecular Engineering, School of Chemistry, IGCME, Sun Yat-sen University, Guangzhou 510275, China; MOE Key Laboratory of Bioinorganic and Synthetic Chemistry, GBRCE for Functional Molecular Engineering, School of Chemistry, IGCME, Sun Yat-sen University, Guangzhou 510275, China; MOE Key Laboratory of Bioinorganic and Synthetic Chemistry, GBRCE for Functional Molecular Engineering, School of Chemistry, IGCME, Sun Yat-sen University, Guangzhou 510275, China; MOE Key Laboratory of Bioinorganic and Synthetic Chemistry, GBRCE for Functional Molecular Engineering, School of Chemistry, IGCME, Sun Yat-sen University, Guangzhou 510275, China; MOE Key Laboratory of Bioinorganic and Synthetic Chemistry, GBRCE for Functional Molecular Engineering, School of Chemistry, IGCME, Sun Yat-sen University, Guangzhou 510275, China

**Keywords:** MOF-based molecular sieve membrane, CO_2_ electroreduction, air, flue gas, pure HCOOH

## Abstract

Efficient electrochemical reduction of CO_2_ (eCO_2_RR) in air and flue gas to high-purity products is crucial for reducing atmospheric CO_2_ levels. However, low CO_2_ concentrations (400 ppm in air, 15% in flue gas) and reducible impurities present significant challenges. To address this, we developed an electrolyzer integrating a self-supporting metal–organic framework-based mixed-matrix molecular sieve membrane (MOF-MMM), a conductive diffusion layer and a Bi nanoparticle catalytic layer. This design enables simultaneous gas purification and eCO_2_RR while avoiding electroreduction of impurities (SO_2_, NO, O_2_). The MOF-MMM enriches CO_2_ from 15% to 82.5% in flue gas or from 0.04% to 2.05% in air. Under acidic conditions, enriched CO_2_ is reduced to formic acid. Using flue gas as feedstock, the eCO_2_RR current reaches 9000 mA with 100% Faradaic efficiency, producing 23 mL of pure HCOOH after 4 h, representing state-of-the-art performance. Using air as feedstock, it achieves 98.2% FE_HCOOH_ with a record partial current density of 5.3 mA cm^−2^ at 2.5 V, yielding 177 200 μmol h^−1^ g^−1^, which is 5000 times higher than reported catalysts without molecular sieves. This integrated approach enables the direct transformation of dilute CO_2_ emissions into transportable liquid fuels, offering dual environmental benefits through carbon utilization and sustainable chemical production.

## Teaser

A self-supporting MOF-based molecular sieve membrane embedded in an electrolyzer enhances CO_2_ electroreduction from air and flue gas to high-purity formic acid efficiently.

## INTRODUCTION

Directly converting CO_2_ from air (400 ppm CO_2_, 21% O_2_, 78% N_2_) into fuel is critical for reducing atmospheric CO_2_ levels and ensuring respiratory safety in confined environments like submarines and space stations [1–4]. Similarly, converting CO_2_ from flue gas (15% CO_2_, 81% N_2_, 4% O_2_, 500 ppm NO and 500 ppm SO_2_) offers a cost-effective and efficient approach to reduce CO_2_ emissions [5,6]. The electrochemical CO_2_ reduction reaction (eCO_2_RR) with renewable or abandoned electricity to convert CO_2_ into value-added chemicals is one of the most promising technologies for achieving carbon neutrality [7]. However, eCO_2_RR faces two main challenges: low CO_2_ concentrations and competitive reactions from gases like O_2_, NO and SO_2_ [8–10], especially in air, where effective reactions are nearly impossible. Moreover, current eCO_2_RR products are either gaseous or liquid mixtures [11–16], requiring costly separation processes for commercial use. For gaseous products, separating CO_2_ is indispensable. For liquid products, even though gas–liquid separation can yield high-purity products, advanced electrolyzers with solid-state electrolytes (SSEs) produce 70%–80% water solutions [17], and further concentration and separation are necessitated. These issues stem from both catalyst performance and electrolyzer design. Therefore, aside from developing new high-performance catalysts, development of new electrolyzers is essential.

**Scheme 1.**
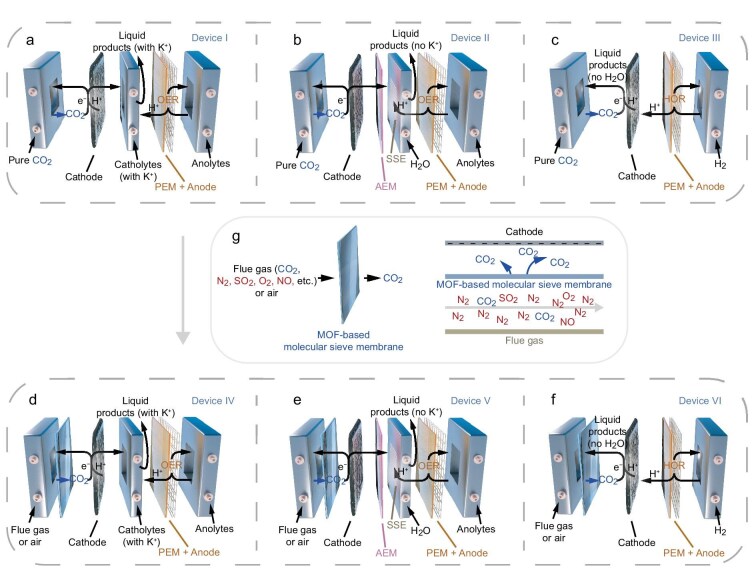
Electrolyzers for eCO_2_RR to HCOOH. The (a) flow cell, (b) MEA-SSE electrolyzer and (c) gas–solid acidic membrane electrolyzer without MOF-based molecular sieve membrane, and (d) flow cell, (e) MEA-SSE electrolyzer and (f) gas–solid acidic membrane electrolyzer embedded with MOF-based MMM in this work. The flow cell for eCO_2_RR to HCOOH was with a liquid electrolyte. The MEA-SSE electrolyzer for eCO_2_RR to electrolyte-free HCOOH was with water. The gas–solid acidic membrane electrolyzer was for eCO_2_RR to anhydrous and electrolyte-free HCOOH. (g) Schematic diagram of the membrane separation device.

Currently, there are three main types of electrolyzers: H-type cells, flow cells (Scheme 1a, Device I) and membrane electrode assembly (MEA) electrolyzers [18–20]. The first two require liquid electrolytes, while MEA electrolyzers can use porous SSEs (denoted as MEA-SSE electrolyzers) (Scheme 1b, Device II). In an MEA-SSE electrolyzer, liquid products can enter the SSE and be flushed away by water or gas, potentially yielding dry, electrolyte-free liquid products, such as formic acid. However, the dense packing of SSE hinders gas flow, leading to high resistance and low flow rates, which increase overpotential and energy waste. Actually, to reduce the resistance, an ion exchange membrane can replace the three layers of AEM, SSE and proton exchange membrane (PEM) to separate the cathode and anode. However, if using an anion exchange membrane (AEM), water molecules on the cathode side are required for proton supply, making it impossible to obtain dry reduced products. In contrast, a PEM without solid and liquid electrolytes allows proton transfer from the anode to reach the cathode (denoted as gas–solid acidic membrane electrolyzer) (Scheme 1c, Device III), enabling formic acid production without a water by-product under acidic conditions. The liquid formic acid can be carried out by a CO_2_ gas stream, resulting in anhydrous, electrolyte-free products. Based on the analysis of the reaction mechanism, eCO_2_RR by a gas–solid acidic membrane electrolyzer must be conducted in the presence of a large number of protons, that is, in strong acidic conditions. In fact, performing eCO_2_RR under acidic conditions has more advantages than under alkaline or neutral conditions, as it can reduce CO_2_ loss to increase the utilization rate of CO_2_ and minimize clogging caused by carbonate deposition [21]. There have been reports in the literature that a composite catalyst composed of In or Sn or Bi metal nanoparticles (NPs) [22–24] and fully perfluorinated polymer [25] can achieve high-performance eCO_2_RR to formic acid under strong acidic conditions. Under these circumstances, the use of such a composite catalyst in gas–solid acidic membrane electrolyzers enables the chance to obtain anhydrous, high-purity and electrolyte-free formic acid.

To directly electrocatalytically reduce CO_2_ in flue gas or air, current strategies involve coating porous materials-based separation membranes (namely, non-self-supporting separation membranes) that adsorb and enrich CO_2_ onto a conductive gas diffusion layer (GDL) or an electrocatalyst-loaded GDL to form multifunctional electrodes (Fig. S1 in Supplementary data) [8,26–28]. However, these non-self-supporting separation membranes can also act as catalysts, reducing not only CO_2_ but also impurities like SO_2_, NO and O_2_ from flue gas and air, which lowers the Faraday efficiency (FE) of CO_2_ reduction. A potential solution is to install a self-supporting CO_2_-selective molecular sieve membrane between the gas inlet and the catalyst-loaded GDL (Fig. S1). This membrane acts purely as a CO_2_ filter without catalytic activity in flow cells (Scheme 1d, Device IV), MEA-SSE electrolyzers (Scheme 1e, Device V) or gas–solid acidic membrane electrolyzers (Scheme 1f, Device VI). This design enables simultaneous gas purification and CO_2_ reduction while avoiding the reduction of gas impurities, offering greater flexibility and universality in optimizing both CO_2_ purification and reduction performance. Mixed matrix membranes (MMMs) combine the processability of organic polymers with the high selectivity of porous inorganic or metal–organic framework (MOF) materials with well-defined pore structures to accommodate various separation application needs [29]. Polymer of intrinsic microporosity (PIM-1), an amorphous polymer with rigid pores, is a cutting-edge material for MMM fabrication thanks to its superior gas permeability [30], yet limited by the low gas selectivity in the practical application. Thereupon, numerous porous materials such as nanofillers have been successfully integrated into PIM-1 or other polymer matrices to fabricate MMMs for selective separations [31,32]. As a kind of crystalline porous material, MOFs have definite pore structures, designability and modifiability and hence have broad applications in the fields of gas storage and separation [33]. We therefore constructed an unprecedented multifunctional electrolyzer by placing an MMM composed of MOF and PIM-1 as the molecular sieve membrane between the gas inlet and GDL loaded with a Bi-based composite catalyst. This unprecedented configuration simultaneously achieves CO_2_ enrichment and improvement of eCO_2_RR performance. Systematic investigations were conducted to elucidate the mechanism and evaluate the performance of direct electroreduction processes using both simulated flue gas and ambient air as feedstock, ultimately producing pure formic acid, even anhydrous, high-purity and electrolyte-free formic acid.

## RESULTS AND DISCUSSION

The Bi NPs were synthesized by a rapid reduction method of NaBH_4_ [34]. Its purity and morphology were confirmed by powder X-ray diffraction (PXRD) patterns (Fig. S2) and high-resolution transmission electron microscopy (HR-TEM) images (Fig. S3). We then prepared the working cathode for eCO_2_RR by coating the microcrystalline Bi NP catalyst on carbon paper as the GDL using (10 wt%) fully perfluorinated polymer as binder (see Supplementary data for details), i.e. Bi/GDL as a working cathode for eCO_2_RR, and IrO_2_ as a working anode for the oxygen evolution reaction (OER). The performance tests were carried out in a traditional two-electrode flow cell (Scheme 1a, Device I) with CO_2_-saturated 0.5 M K_2_SO_4_ + 0.05 M H_2_SO_4_ aqueous solution (pH 1) as electrolyte (see Supporting data for details). The concentration of HCOOH was measured by ^1^H nuclear magnetic resonance (^1^H NMR) spectra (Fig. S4). From the linear sweep voltammetry (LSV) curve (Fig. S5a), it can be seen that the platform disappeared above a cell potential of 2.5 V, and there was a significant increase in current with the increase of potential, which may be due to the acceleration of HCOOH formation. Subsequently, we conducted potentiostatic electrolysis at different potentials from 2.5 to 4.5 V (Fig. S5b) and analyzed different products. The ^1^H NMR spectra (Fig. S5c and d) showed that as the potential increases, the FE_HCOOH_ value is always greater than 95%, and the current density reaches its maximum of 0.84 A cm^−2^ at a cell potential of 4.3 V, giving a spatiotemporal yield rate of up to 1.4 g h^−1^ mg_cat_^−1^, representing one of the best performance levels currently documented (Table S1). Besides, the FE_HCOOH_ value can maintain 82.8% with the ampere level current density of 1.04 A cm^−2^ at a cell potential of 4.5 V. The above results indicate that the composite catalyst consisting of Bi NPs and fully perfluorinated polymer enables high-performance eCO_2_RR to produce HCOOH under strong acid conditions and is suitable for working in a gas–solid acidic membrane electrolyzer (Scheme 1c and f).

To assess the performance of eCO_2_RR under flue gas conditions, we chose a typical flue gas (15% CO_2_, 81% N_2_, 4% O_2_, 500 ppm NO and 500 ppm SO_2_) as feedstock for eCO_2_RR in a traditional two-electrode flow cell (Scheme 1a, Device I) with a CO_2_-saturated 0.5 M K_2_SO_4_ + 0.05 M H_2_SO_4_ aqueous solution as electrolyte. Due to the presence of the competitive O_2_ reduction reaction (ORR), NO reduction reaction (NORR) and SO_2_ reduction reaction (SO_2_RR), the possible reduction products H_2_O_2_ and NH_3_ were measured by a chronoamperometric method (Figs S6 and S7). In the same potential range of 2.5–4.5 V, the results of ^1^H NMR and UV-vis measurements show that when using flue gas as the feedstock, instead of pure CO_2_, the NORR product NH_3_ was detected, while the ORR product H_2_O_2_ was not detected (Fig. S8b and c). Using O_2_/N_2_ (20/80) as the feedstock and Bi/GDL as the working cathode, it is evident that the hydrogen evolution reaction (HER) dominates, while no ORR product H_2_O_2_ is detected (Figs S10 and S11). This indicates that the Bi NP catalysts exhibit no ORR activity under acidic conditions. Consequently, the absence of H_2_O_2_ product during eCO_2_RR under flue gas conditions can be attributed to the lack of ORR activity by the Bi NP catalyst under acidic conditions. From the LSV curves (Fig. S9a), it is observable that the current density under flue gas conditions is significantly lower than that under pure CO_2_ conditions throughout the entire potential range. The highest FE_HCOOH_ value under flue gas conditions is 59.4% ± 3.0% at 2.5 V (Fig. S9c), which is also notably lower than the 99.2% ± 0.4% obtained under pure CO_2_ conditions. Considering the reduction products of eCO_2_RR and NORR, along with HER, the total FE value remains far below 100% under flue gas conditions, indicating the existence of other side reactions. The X-ray photoelectron spectroscopy (XPS) spectra and element analysis (EA) showed that there was 5.28 wt% sulfur (Fig. S8d and Table S2) in the catalyst after the electrochemical test, suggesting that SO_2_ was also reduced. The aforementioned experimental results indicate that the main reason for the decrease in performance (current density and FE value) is the lower concentration of CO_2_, as well as the competitive reduction reaction of SO_2_ and NO.

**Figure 1.**
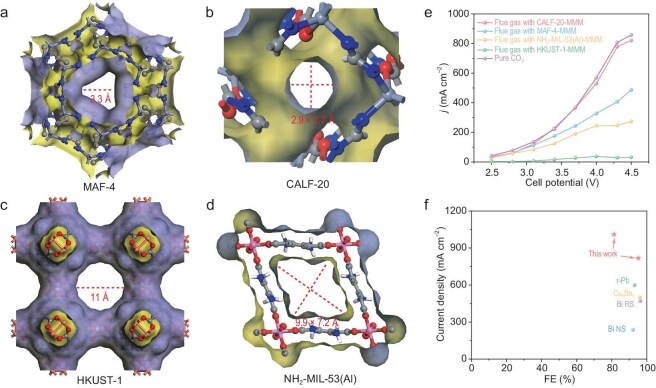
Pore structures of different MOFs: (a) MAF-4, (b) CALF-20, (c) HKUST-1 and (d) NH_2_-MIL-53(Al). (e) The partial current densities of HCOOH at different potentials by Bi NPs as catalyst with flue gas as feedstock in flow cells embedded with different MOF-MMM as molecular sieve membranes. (f) Comparison of current density and FE of HCOOH for representative electrocatalysts for eCO_2_RR to produce HCOOH (Table S1).

A metal–azolate framework (MAF) [Zn(mim)_2_] (MAF-4, also known as ZIF-8, where mim = 2-methylimidazolate) with a solidate (SOD) topology (Fig. S12) features nanosized cages with six-membered ring apertures of an accessible size of 3.3 Å (Fig. 1a) [35]. Possessing advantages such as high stability, straightforward synthesis, high hydrophobicity and excellent gas separation performance, MAF-4 distinguishes itself in membrane separations, and has been utilized for the adsorption and separation of the mixture of CO_2_ and N_2_ [36]. Therefore, MAF-4 and PIM-1 were used to fabricate MAF-4-MMM as a molecular sieve membrane in the electrolyzer for purification of the low concentration CO_2_ from flue gas. MAF-4 was prepared by simple stirring at room temperature, and its purity was confirmed by PXRD patterns (Fig. S13) and thermogravimetric (TG) curves (Fig. S14). The gas sorption isotherms of MAF-4 measured at 298 K (Fig. S15a) showed a CO_2_ adsorption capacity of 0.67 mmol g^−1^, while those of N_2_, O_2_ and SO_2_ are just 0.08, 0.09 and 0.18 mmol g^−1^, respectively, being similar to the reported results [37–39]. As shown in the pore size distribution curve of PIM-1 presented in Fig. S15c, the micropore distributions (1.2–1.3 nm) are in agreement with literature reports [40]. The selectivity of CO_2_/N_2_, CO_2_/O_2_ and CO_2_/SO_2_ was calculated as 8.4, 7.4 and 3.7, respectively. Above results indicate that MAF-4-MMM is possible to achieve purification of the low concentration CO_2_ from flue gas. MAF-4-MMM was fabricated by a blending method (see Supplementary data for details), and the PXRD pattern showed that the structure of MAF-4 was maintained after membrane formation (Fig. S13). Scanning electron microscopy (SEM) images showed that MAF-4 is uniformly distributed in MAF-4-MMM with a thickness of 70 μm and the surface of MAF-4-MMM is smooth (Fig. S16).

To evaluate the filtration performance of the MOF-MMM in the electrochemical flue gas reduction process, we placed MAF-4-MMM between the gas inlet and the GDL loaded with Bi-based composite catalysts in a two-electrode flow cell (Scheme 1d, Device IV), using CO_2_-saturated 0.5 M K_2_SO_4_ + 0.05 M H_2_SO_4_ aqueous solution (pH 1) as electrolyte, Bi/GDL as a working cathode for eCO_2_RR, and IrO_2_ as a working anode for OER. Interestingly, as shown in Fig. S17b and c, there were no NH_3_ and H_2_O_2_ present in the products, indicating that the MAF-4-MMM does act as an *in situ* barrier against NO. Also, the EA result showed there is no sulfur in the catalyst, suggesting no SO_2_ reduction (Table S2). It can be seen that the current density with MAF-4-MMM is higher than that without MAF-4-MMM, and FE_HCOOH_ can maintain above 80% within the whole voltage range (Fig. S18a). The composition analysis of flue gas passing through MAF-4-MMM (Figs S20–S22) reveals effective blockage of N_2_, O_2_, SO_2_ and NO, while CO_2_ exhibits selective permeation at 3.8 mL min^−1^ cm^−2^. This results in CO_2_ concentration enhancement from 15% to 81.7% (N_2_: 17.0%; O_2_: 1.3%; SO_2_: 23.1 ppm; NO: 15.0 ppm). The observed selectivity stems from the smaller aperture size of MAF-4, which excludes SO_2_ (dynamic radius of 3.6 Å) over CO_2_ (3.3 Å). Furthermore, the weak MAF-4 interactions with NO (3.17 Å), analogous to its interactions with N_2_ (3.64 Å) and O_2_ (3.46 Å), synergistically enhance the gas separation. Through combined molecular sieving (kinetic) and affinity adsorption (thermodynamic) selectivity mechanisms, the MAF-4-MMM in the electrolyzer effectively filters O_2_, NO, SO_2_ and N_2_ from flue gas, thereby delivering purified CO_2_ to the working cathode. Notably, although using MAF-4-MMM, the current density with flue gas as feedstock (599 mA cm^−2^) is still lower than that under pure CO_2_ conditions (1040 mA cm^−2^). We speculated that the flow rate of CO_2_ passing through MAF-4-MMM may not be sufficient to meet the CO_2_ consumption in eCO_2_RR at higher current density. Specifically, at a current density of 599 mA cm^−2^ with a cell potential of 4.5 V, the consumption rate of CO_2_ is 3.7 mL min^−1^ cm^−2^. Considering the conversion rate of 97.4%, the amount of CO_2_ (3.8 mL min^−1^ cm^−2^) provided by the MAF-4-MMM indeed cannot meet the CO_2_ demand of the eCO_2_RR on the catalytic layer (i.e. the so-called supply falling short of demand), resulting in a relatively small current density.

To improve the performance, we then utilized Calgary framework 20 (CALF-20) (Fig. 1b), which features a smaller aperture size of 2.7 × 2.9 Å and exhibits a higher CO_2_ adsorption capacity compared to MAF-4, for the selective adsorption of CO_2_ from flue gas [41]. CALF-20 was prepared according to the literature method, and its purity was confirmed by PXRD patterns (Fig. S23) and TG curves (Fig. S24). The gas sorption isotherms of CALF-20 at 298 K (Fig. S25) show a CO_2_ adsorption capacity of 3.62 mmol g^−1^, while those of N_2_ and O_2_ are just 0.29 and 0.08 mmol g^−1^, respectively. CALF-20-MMM was obtained by a blending method similar to that for MAF-4-MMM. SEM images showed that CALF-20 is uniformly distributed in MMM with a thickness of 70 μm and its surface is smooth (Fig. S26). To evaluate the filtration performance of CALF-20-MMM in the electrochemical flue gas reduction process, we conducted tests under the same conditions (Scheme 1d, Device IV), except for replacing MAF-4-MMM by CALF-20-MMM. When using CALF-20-MMM, there are no NH_3_ and H_2_O_2_ present in the products (Fig. S27b and c), while the EA result shows no sulfur in the catalyst (Table S2), indicating that CALF-20-MMM also acts as an *in situ* barrier against NO and SO_2_. Interestingly, the eCO_2_RR current density by Bi/GDL in flow cell embedded with CALF-20-MMM (1010 mA cm^−2^) is higher than that embedded with MAF-4-MMM (599 mA cm^−2^) and very close to that by Bi/GDL in pure CO_2_ (1040 mA cm^−2^) (Fig. S28a). Also, the FE_HCOOH_ can be maintained above 95% within the potential range of 2.5–4.3 V (Fig. S28c) and the current density reaches its maximum of 0.82 A cm^−2^ at a cell potential of 4.3 V. Besides, the FE_HCOOH_ value can be maintained at 81.3% with the ampere-level current density of 1.01 A cm^−2^ at a cell potential of 4.5 V with 83.4% single-pass conversion efficiency for CO_2_ at a very high gas flow rate of 50 mL  min^−1^, representing state-of-the-art performance (Fig. 1f and Table S1), demonstrating CALF-20-MMM can enrich low concentration CO_2_  *in situ* in flue gas and the supply of CO_2_ can meet the needs in the high cell potential for eCO_2_RR. By analyzing the gas compositions of flue gas passing through CALF-20-MMM (Figs S21, S22 and S29), it is evident that N_2_, O_2_, SO_2_ and NO are effectively blocked, while CO_2_ selectively permeates with a much higher flow rate of 7.3 mL min^−1^ cm^−2^ by using CALF-20-MMM than by using MAF-4-MMM. Consequently, the CO_2_ concentration increased from 15% to 82.5% (N_2_: 16.4%; O_2_: 1.1%; SO_2_: 26.3 ppm; NO: 14.4 ppm). At a current density of 1010 mA cm^−2^ with a cell potential of 4.5 V, the consumption rate of CO_2_ is 6.3 mL min^−1^ cm^−2^, meaning that the supply meets the consumption requirements. When CALF-20-MMM was replaced by just the PIM-1 membrane without MOF materials (Fig. S30), the reduction products of SO_2_RR and NORR were still detected (Fig. S31 and Table S2). The compositions of products were basically consistent with those without the PIM-1 membrane in a traditional two-electrode flow cell (Scheme 1a, Device I) under flue gas conditions (Fig. S32), demonstrating that all gas components in the flue gas could pass through the PIM-1 membrane. In the absence of Bi NPs as a catalyst, no eCO_2_RR product was detected, while hydrogen gas was the sole product (Figs S33 and S34). This fact indicates that the molecular sieve membrane in the electrolyzer acts solely as a filter due to no electrical continuity between CALF-20-MMM and GDL. According to the literature method [28], when CALF-20 microcrystal powder was directly coated on the GDL gas inlet side (Fig. S1, namely, non-self-supporting separation membranes), forming a Bi/GDL/CALF-20 working cathode, products include CO, formic acid and NH_3_ from NORR (Figs S35 and S36), indicating catalytic activity from both Bi NPs and CALF-20. These results show that MOFs with CO_2_ enrichment capabilities, when directly coated on GDL, can also catalyze undesired side reactions. The findings demonstrate that: (i) MOF-MMMs with PIM-1 membranes effectively filter N_2_, O_2_, NO and SO_2_ from flue gas; (ii) when CALF-20-MMM is fabricated between the gas inlet and the GDL in an electrolyzer, only Bi NPs are catalytically active; and (iii) compared to the methods of direct MOF coating on GDL reported in the literature, MOF-MMMs achieve CO_2_ enrichment without causing side reactions.

**Figure 2.**
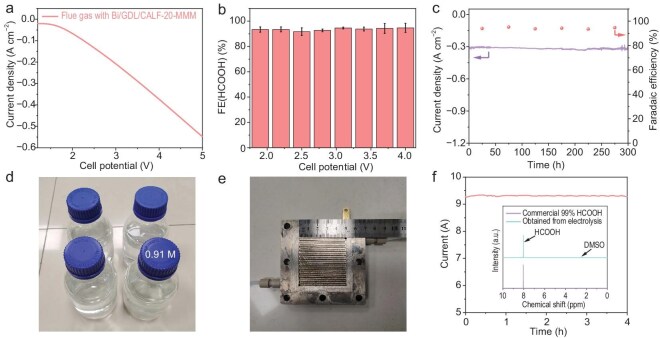
Performance of eCO_2_RR by Bi-based catalyst with flue gas as feedstock in MEA-SSE electrolyzer embedded with CALF-20-MMM. (a) LSV curve. (b) FE of HCOOH. (c) Stability test over 300 h in electrocatalysis at the potential of 4.0 V. (d) 1.8 L of HCOOH aqueous solution (0.91 M). (e) Photograph of cathode flow field of 25-cm^2^ gas–solid electrolyzer of MEA. (f) 4-h *i–t* curve with current of 9 A; insert shows ^1^H NMR spectrum of commercial 99% HCOOH and that obtained from electrolysis.

By virtue of the filtration performance of CALF-20-MMM, the eCO_2_RR performances in flue gas were also evaluated in an MEA-SSE electrolyzer embedded with CALF-20-MMM (Scheme 1e, Device V) to yield pure HCOOH aqueous solution without salt with Bi/GDL as a working cathode for eCO_2_RR and Pt/C (5 wt%) as a working anode for the hydrogen oxidation reaction (HOR). They were separated by AEM, SSE and PEM. Under the electric field, the generated HCOO^−^ from the cathode was combined with H^+^ ions from the anode and migrated through the SSE layer to yield HCOOH molecules, which were then discharged via a deionized water stream to obtain HCOOH aqueous solution. Notably, flue gas was used as the feedstock, while no cathodic and anodic electrolyte was used in the MEA of the CO_2_//SSE//H_2_ electrolyzer. The LSV curve showed that the platform appeared before a cell potential of 1.9 V, and there was a significant increase in current within the range of 1.9–4.0 V, which may be due to the acceleration of HCOOH formation (Fig. 2a). We then conducted potentiostatic electrolysis at different potentials from 1.9 to 4.0 V to evaluate the eCO_2_RR performance. The ^1^H NMR spectra (Fig. S37a) showed that FE_HCOOH_ can be maintained above 90% within the whole voltage range and achieve the highest current density of 340 mA cm^−2^ at 4.0 V. To evaluate the stability, a continuous eCO_2_RR was performed at 4.0 V. The result (Fig. 2c) indicated that the working cathode can continuously work for at least 300 h at a high yield rate of 495.1 mg h^−1^ mg_cat_^−1^, giving 1.8 L of 0.91 M HCOOH aqueous solution without salt (Fig. 2d and Fig. S38), which represents exceptional performance (Table S3). According to the TEM images (Fig. S39), XPS spectra (Fig. S40) and PXRD patterns (Fig. S2), the working cathode remained nearly unaltered after electrolysis, suggesting the catalytic system is highly stable during electrocatalysis.

To obtain pure and anhydrous HCOOH, the eCO_2_RR performance in flue gas was evaluated in a gas–solid acidic membrane electrolyzer embedded with CALF-20-MMM (Scheme 1f, Device VI) with a Bi/GDL as a working cathode for eCO_2_RR and Pt/C (5 wt%) as a working anode for HOR. They were separated by just the PEM for efficient ion transportation. Under the electric field, the H^+^ ions generated from the anode migrated to the cathode through the PEM and combined with HCOO^−^ generated from the cathode to yield HCOOH molecules, which were purged from the flue gas stream and trapped to obtain pure HCOOH. To achieve this scenario, an MEA with a window area of 5 × 5 cm^2^ and without any electrolyte was used with a catalyst mass of 12.5 mg (Fig. 2e). After 4 h of electrolysis with a current of 9 A and flue gas as feedstock, we successfully collected 23 mL (28 g) of electrolyte-free anhydrous formic acid of analytical grade, and the ^1^H NMR spectrum showed the absence of H_2_O (Fig. 2f). These results demonstrate the effectiveness of this method for directly obtaining pure formic acid under flue gas conditions. This is the first report of eCO_2_RR directly yielding an analytical-grade anhydrous product, which establishes the groundwork for the industrial application of eCO_2_RR directly to yield commercial reagents in the future.

To further verify the differences in the performance originating from the adsorption and separation effects of MOFs in MMMs on different gases in flue gas, Hong Kong University of Science and Technology 1 (HKUST-1) and NH_2_-MIL-53(Al) with larger aperture sizes of 11 (Fig. 1c) and 9.9 × 7.2 Å (Fig. 1d), respectively, were selected. Their purities were confirmed by PXRD patterns (Fig. S41). The sorption isotherms of HKUST-1 at 298 K (Fig. S42a) show a CO_2_ adsorption capacity of 2.89 mmol g^−1^, while those of N_2_ are just 0.22 mmol g^−1^. The sorption isotherms of NH_2_-MIL-53(Al) at 298 K (Fig. S42b) show a CO_2_ adsorption capacity of 2.17 mmol g^−1^, while those of N_2_ just 0.01 mmol g^−1^. HKUST-1-MMM and NH_2_-MIL-53(Al)-MMM were obtained by a similar blending method and the SEM images show the successful preparation (Figs S41, S43 and S44). Their performances were evaluated with flue gas as feedstock under the same conditions (Scheme 1d, Device IV), except for replacing MAF-4-MMM by NH_2_-MIL-53(Al)-MMM and HKUST-1-MMM, respectively. Under flue gas conditions, the results using HKUST-1-MMM are similar to those without MMM (Fig. S46). The products contain NH_3_ (Fig. S45b) and sulfur element (Table S2), indicating that all gas components in the flue gas can pass through HKUST-1-MMM. When using NH_2_-MIL-53(Al)-MMM, the current density is similar to that using MAF-4-MMM (Fig. S48a), but the FE_HCOOH_ is much lower (Fig. S48c). By analyzing the gas compositions of flue gas passing through HKUST-1-MMM and NH_2_-MIL-53(Al)-MMM (Fig. S49), it can be seen that CO_2_, O_2_ and N_2_ pass through, giving a flow rate of 11.0 and 9.7 mL min^−1^ cm^−2^, respectively. Consequently, the CO_2_ concentrations were just increased from 15% to 46.5% and 43.6%, respectively. Since the pore sizes of HKUST-1 and NH_2_-MIL-53(Al) are much larger than the dynamic radii of O_2_, N_2_, NO and SO_2_, these MOFs cannot function as molecular sieves to filter out O_2_, N_2_, NO and SO_2_. From the above results, it is evident that the aperture sizes of MOFs and their CO_2_ adsorption capacities at 0.15 bar can significantly affect the enrichment performance of MOF-MMM for CO_2_ in flue gas, which in turn affects the performance of the electrolysis. Appropriate molecular sieve membranes can effectively enhance the direct electroreduction of CO_2_ in flue gas in an acidic membrane electrolyzer.

**Figure 3.**
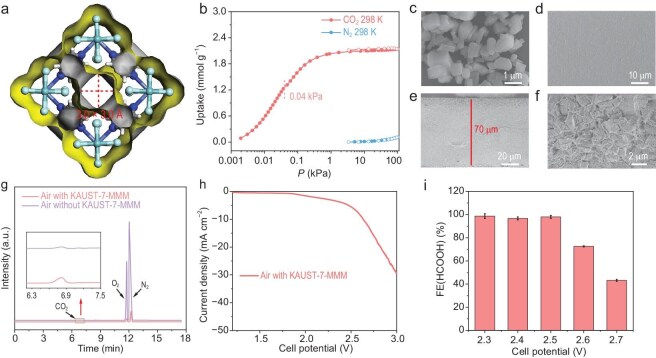
Structure, CO_2_ capture and eCO_2_RR performances of KAUST-7. (a) Pore structure of KAUST-7. (b) CO_2_ and N_2_ sorption isotherms of KAUST-7 at 298 K. The marked adsorption capacity of CO_2_ is 1.25 mmol g^−1^ at 0.04 kPa of KAUST-7 measured at 298 K. (c) SEM image of KAUST-7. (d) SEM image of the surface of KAUST-7-MMM. (e and f) SEM images of cross-section of KAUST-7-MMM at different resolutions. (g) Gas chromatography profiles of air passing through KAUST-7-MMM. (h) LSV curve and (i) FE_HCOOH_ by Bi NPs as catalyst with air as feedstock in flow cell embedded with KAUST-7-MMM.

Furthermore, the eCO_2_RR performance of Bi/GDL was also evaluated by air as the feedstock in a flow cell embedded with CALF-20-MMM (Scheme 1d, Device IV). The CO_2_-saturated 0.5 M K_2_SO_4_ + 0.05 M H_2_SO_4_ aqueous solution (pH 1) was used as electrolyte. However, no reduction products of eCO_2_RR were detected (Fig. S50). This may mainly be ascribed to the fact that CALF-20 hardly adsorbs CO_2_ at a concentration of 400 ppm (Fig. S25). From the aforementioned results, to achieve efficient electroreduction of CO_2_ with a concentration as low as 400 ppm in air, the MOF in the molecular sieve membrane must be capable of highly efficient CO_2_ capture from air. With an aperture size of 3.0 × 3.0 Å, King Abdullah University of Science and Technology 7 (KAUST-7) (Fig. 3a) has been demonstrated to exhibit superior CO_2_ capture performance from air through breakthrough experiments [42,43]. Also, a KAUST-7-MMM can effectively enrich CO_2_ from a mixed gas CO_2_/N_2_ (15/85) [44]. However, KAUST-7-MMM for the separation and enrichment of CO_2_ from air has not yet been studied. According to the literature, KAUST-7 was synthesized, and its purity was confirmed by PXRD pattern (Fig. S51). The CO_2_ sorption isotherms at 298 K showed that the adsorption capacity of CO_2_ of KAUST-7 is 2.14 mmol g^−1^ at 100 kPa, while the adsorption capacity of CO_2_ is as high as 1.25 mmol g^−1^ at 0.04 kPa (Fig. 3b); those are similar to the reported results [45]. KAUST-7-MMM was obtained using a blending method similar to that for MAF-4-MMM. SEM images showed that KAUST-7 is uniformly distributed in MMM with a thickness of 70 μm and its surface is smooth (Fig. 3c–f). By analyzing the gas compositions of air passing through the KAUST-7-MMM, it is evident that the concentrations of O_2_ and N_2_ were significantly reduced, while that of CO_2_ increased from 0.04% to 2.05% (N_2_: 76.98%; O_2_: 20.97%), representing a 50-fold increase (Fig. 3g). The CO_2_ permeates through the molecular sieve membrane with a flow rate of 3.3 mL h^−1^ cm^−2^. To our knowledge, this is the first molecular sieve membrane that can enrich CO_2_ from the air. When using Bi/GDL as the working cathode for eCO_2_RR, and IrO_2_ as the working anode for OER, eCO_2_RR was conducted in a flow cell embedded with KAUST-7-MMM (Scheme 1d, Device IV). The CO_2_-saturated 0.5 M K_2_SO_4_ + 0.05 M H_2_SO_4_ aqueous solution (pH 1) was used as electrolyte. As shown in Fig. 3h and i, when directly using air as the feedstock, it achieved an FE_HCOOH_ of 98.2% with a maximum partial current density of 5.3 mA cm^−2^ at 2.5 V (Fig. S53), giving a high and record yield rate of 177 200 μmol h^−1^ g^−1^. Such high performance by Bi/GDL in flow cell embedded with KAUST-7-MMM is at least 5000 times higher than those of reported catalysts without a molecular sieve membrane (Table S4), demonstrating the important role of the MOF-based molecular sieve membrane during the eCO_2_RR process. When using a gas–solid acidic membrane electrolyzer (window area of 10 × 10 cm^2^) embedded with KAUST-7-MMM (Scheme 1f, Device VI), after 300 h of electrolytic treatment, 32.6 L of CO_2_ were removed from the air, resulting in 50.2 mL of pure formic acid (Fig. S54). As evidenced by the PXRD pattern (Fig. S51) and SEM images (Fig. S55) of KAUST-7-MMM after electrocatalytic operation, the diffraction peaks of KAUST-7 remained intact and its uniform distribution within the membrane was preserved, thereby confirming the structural and morphological stability of the MMM. Considering the remarkable one-step CO_2_ capture and conversion capability of such an electrolyzer embedded with KAUST-7-MMM, which is both simple and efficient, we anticipate that this technology holds significant potential for future applications in confined environments such as submarines and space stations.

## CONCLUSIONS

In summary, to our knowledge for the first time, we have demonstrated that an acidic membrane electrolyzer embedded with self-supporting MOF molecular sieve membranes can not only directly enrich CO_2_ from flue gas and air to produce purified and concentrated CO_2_, but also directly catalyze it to yield pure formic acid. Also, importantly, the obtained pure formic acid product does not contain any electrolyte and is anhydrous, which can directly meet commercial needs. Given that this electrolytic device can flexibly adjust the catalytic layer and molecular sieve layer, and significantly reduce the cost of capturing CO_2_ from flue gas and air, as well as the cost of product separation, this study provides a new research perspective for the efficient and low-energy-consumption utilization of CO_2_ directly from air and flue gas.

## METHODS

### Synthesis of Bi NPs

Polyvinyl pyrrolidone (300 mg) and Bi(NO_3_)_3_·5H_2_O (100 mg) were dissolved in a mixture of 10 mL of glycerol and 5 mL of ethanol, which had been saturated with Ar for 30 min. Subsequently, 50 mg of NaBH_4_ was rapidly added to the solution, followed by stirring at 30°C for 1 min. After the reaction, the products were collected by centrifugation at 14 750 ×g for 20 min and washed three times sequentially with anhydrous ethanol and deionized water. Finally, the products were dried under vacuum at 60°C.

### Synthesis of PIM-1

A mixture of 8.32 g of K_2_CO_3_, 10.25 g of 3,3,3′,3′-tetramethyl-1,1′′-spirobisindane-5,5′,6,6′-tetrol and 6.02 g of 2,3,5,6-tetrafluorophthalonitrile was added to 200 mL of dry dimethylformamide and stirred at 65°C for 72 h under N_2_ atmosphere. The mixture was then added to 300 mL of H_2_O and the crude product was collected by filtration.

### Fabrication of MAF-4-MMM

Firstly, 200 mg of pure PIM-1 was dissolved in 10 g of CHCl_3_ with stirring for 1 h to form a homogeneous solution, then 40 mg of MAF-4 was added to the above solution and vigorously stirred for 5 h. After adding 30 mL of methanol, the yellow powder MAF-4/PIM-1 was collected by filtration. After washing with MeOH, filtering and drying, MAF-4/PIM-1 was dissolved in 10 g of CHCl_3_ and then cast in a mold. The obtained MAF-4-MMM after solvent evaporation was soaked in methanol for 24 h to activate MAF-4 and then dried at 80°C overnight. The fabrication procedures for CALF-20-MMM, HKUST-1-MMM, NH_2_-MIL-53(Al)-MMM and KAUST-7-MMM were similar to that of MAF-4-MMM, except that CALF-20, HKUST-1, NH_2_-MIL-53(Al) and KAUST-7 were used instead of MAF-4.

### Electrochemical measurements

Electrochemical measurements were carried out in a typical two-electrode flow cell; an IrO_2_-loaded titanium mesh was employed as the counter electrode. The anolyte and catholyte chambers were separated by a cation exchange membrane (Nafion 115, DuPont). The cathode and anode were connected with copper tape (current collector). For electrochemical CO_2_ reduction, potentiostatic tests at potentials of 2.5, 2.8, 3.1, 3.4, 3.7, 4.0, 4.3 and 4.5 V (without current–resistance compensation) were carried out in CO_2_-saturated 0.05 M H_2_SO_4_ and 0.5 M K_2_SO_4_ mixed solution (the volume of the electrolyte in the anode and cathode chamber was 35 mL for each) for 1 h, which circulated through the cathode and anode at a rate of 10 mL min^−1^ under the pressure applied by a peristaltic pump (EC200-01, Gauss Union). The constant-potential electrolysis experiments were repeated three times, and the results presented are the averaged values. The CO_2_ flow was kept constant at 50 sccm using a flow controller (KMG3, Alicat Scientific). The effective area of the working electrode in the flow cell was 1 × 1 cm^2^.

## Supplementary Material

nwaf329_Supplemental_File
