# Supplementary material for: Embedding a self-supporting MOF-based molecular sieve membrane into an electrolyzer for boosting electroreduction of CO2 in air and flue gas to HCOOH
Source: Natl Sci Rev. 2025 Aug 12;12(10):nwaf329. doi: 10.1093/nsr/nwaf329 (PMC12485609; doi:10.1093/nsr/nwaf329)
Supplement: nwaf329_Supplemental_File [file nwaf329_supplemental_file.pdf]

Supplementary information for

Embedding a self-supporting MOF-based molecular sieve membrane into an electrolyzer for boosting electroreduction of CO_2 in air and flue gas to HCOOH

Da-Shuai Huang[1,#], Yu Wang[2,#], Yi Tang[1,#], Jia-Run Huang[1], Pei-Xian Li[1], Cheng-Peng Liang[1], Zhen-Hua Zhao[1], Pei-Qin Liao[1]*, Xiao-Ming Chen[1]

[1]MOE Key Laboratory of Bioinorganic and Synthetic Chemistry, GBRCE for Functional Molecular Engineering, School of Chemistry, IGCME, Sun Yat-Sen University, Guangzhou, 510275, China.

[2]Key Laboratory of Special Functional and Smart Polymer Materials of Ministry of Industry and Information Technology, Xi'an Key Laboratory of Functional Organic Porous Materials, School of Chemistry and Chemical Engineering, Northwestern Polytechnical University, Xi'an, Shaanxi 710072, P.R. China

[#]These authors contributed equally to this work.

*Corresponding author. E-mail: liaopq3@mail.sysu.edu.cn

Table of Contents

Supplementary Figure 1. Comparison between the flow cells.
Supplementary Figure 2. PXRD characterization of Bi NPs.
Supplementary Figure 3. HR-TEM images of Bi NPs.
Supplementary Figure 4. Measurements of the HCOOH concentrations by ^{1}H NMR spectroscopy.
Supplementary Figure 5. Performance of eCO_2RR with pure CO_2 as feedstock by Bi NPs.
Supplementary Figure 6. UV-vis measurements of the concentrations of NH_3.
Supplementary Figure 7. UV-vis measurements of the concentrations of H_2O_2.
Supplementary Figure 8. ^{1}H NMR and UV-vis measurements of products by Bi/GDL with flue gas as feedstock.
Supplementary Figure 9. Performance of eCO_2RR by Bi/GDL with flue gas as feedstock.
Supplementary Figure 10. GC and UV-vis measurements of products by Bi/GDL with O_2/N_2 (20/80) as feedstock.
Supplementary Figure 11. Performance of eCO_2RR by Bi/GDL with O_2/N_2 (20/80) as feedstock.
Supplementary Figure 12. Schematic diagram of **MAF-4**.
Supplementary Figure 13. PXRD characterization of **MAF-4.**
Supplementary Figure 14. Thermogravimetric curve characterization of **MAF-4**.
Supplementary Figure 15. CO_2, N_2, SO_2 and O_2 sorption isotherms of **MAF-4** at 298 K.
Supplementary Figure 16. SEM images of **MAF-4** and **MAF-4**-MMM.
Supplementary Figure 17. ^{1}H NMR and UV-vis measurements of products by Bi/GDL with flue gas as feedstock in flow cell with **MAF-4**-MMM.
Supplementary Figure 18. Performance of eCO_2RR by Bi/GDL with flue gas as feedstock in flow cell with **MAF-4**-MMM.
Supplementary Figure 19. GC profiles of different concentrations of CO_2.
Supplementary Figure 20. GC profiles of CO_2.
Supplementary Figure 21. UV-vis measurements of the concentrations of SO_2.
Supplementary Figure 22. UV-vis measurements of the concentrations of NO.
Supplementary Figure 23. PXRD characterization of **CALF-20**.
Supplementary Figure 24. TGA characterization of **CALF-20**.
Supplementary Figure 25. CO_2, N_2 and O_2 sorption isotherms of **CALF-20** at 298K.
Supplementary Figure 26. SEM images of **CALF-20** and **CALF-20**-MMM.
Supplementary Figure 27. ^{1}H NMR and UV-vis measurements of products by Bi/GDL with flue gas as feedstock in flow cell with **CALF-20**-MMM.
Supplementary Figure 28. Performance of eCO_2RR by Bi/GDL with flue gas as feedstock in flow cell with **CALF-20**-MMM.
Supplementary Figure 29. GC profiles of CO_2.
Supplementary Figure 30. SEM images of PIM-1 membrane.
Supplementary Figure 31. ^{1}H NMR and UV-vis measurements of products by Bi/GDL with flue gas as feedstock in flow cell with PIM-1 membrane.
Supplementary Figure 32. Performance of eCO_2RR by Bi/GDL with flue gas as feedstock in flow cell with PIM-1 membrane.
Supplementary Figure 33. ^{1}H NMR, GC and UV-vis measurements of products by GDL with flue gas as feedstock in flow cell with **CALF-20**-MMM.
Supplementary Figure 34. Performance of eCO_2RR by GDL with flue gas as feedstock in flow cell with **CALF-20**-MMM.

Supplementary Figure 35. ^{1}H NMR, GC and UV-vis measurements of products by Bi/GDL/**CALF-20** with flue gas as feedstock in flow cell.
Supplementary Figure 36. Performance of eCO_2RR by Bi/GDL/**CALF-20** with flue gas as feedstock in flow cell.
Supplementary Figure 37. ^{1}H NMR measurements and *i-t* curves by Bi/GDL with flue gas as feedstock in MEA-SSE electrolyzer with **CALF-20**-MMM.
Supplementary Figure 38. ^{1}H NMR measurements after 300 h electrocatalysis.
Supplementary Figure 39. HR-TEM image and EDS of Bi NPs after electrocatalysis.
Supplementary Figure 40. XPS characterization of Bi NPs.
Supplementary Figure 41. PXRD characterization of **HKUST-1**, **NH_2-MIL-53(Al)**, and their MMMs.
Supplementary Figure 42. CO_2 and N_2 sorption isotherms of **HKUST-1** and **NH_2-MIL-53(Al)** measured at 298 K.
Supplementary Figure 43. SEM images of **HKUST-1** and **HKUST-1**-MMM.
Supplementary Figure 44. SEM images of **NH_2-MIL-53(Al)** and **NH_2-MIL-53(Al)**-MMM.
Supplementary Figure 45. ^{1}H NMR and UV-vis measurements of products by Bi/GDL with flue gas as feedstock in flow cell with **HKUST-1**-MMM.
Supplementary Figure 46. Performance of eCO_2RR by Bi/GDL with flue gas as feedstock in flow cell with **HKUST-1**-MMM.
Supplementary Figure 47. ^{1}H NMR and UV-vis measurements of products by Bi/GDL with flue gas as feedstock in flow cell with **NH_2-MIL-53(Al)** MMM.
Supplementary Figure 48. Performance of eCO_2RR by Bi/GDL with flue gas as feedstock in flow cell with **NH_2-MIL-53(Al)**-MMM.
Supplementary Figure 49. GC profiles of CO_2.
Supplementary Figure 50. Performance of eCO_2RR by Bi/GDL with air as feedstock in flow cell with **CALF-20**-MMM.
Supplementary Figure 51. PXRD characterization of **KAUST-7**.
Supplementary Figure 52. ^{1}H NMR measurements of HCOOH by Bi/GDL with air as feedstock in flow cell with **KAUST-7**-MMM.
Supplementary Figure 53. Performance of eCO_2RR by Bi/GDL with air as feedstock in flow cell with **KAUST-7**-MMM.
Supplementary Figure 54. Stability test.
Supplementary Figure 55. SEM images of **KAUST-7**-MMM after electrocatalysis.
Supplementary Table 1. Comparison of eCO_2RR performances of different catalysts in flow cell.
Supplementary Table 2. Element analysis of Bi NPs after electrocatalysis at different eCO_2RR conditions.
Supplementary Table 3. Comparison of eCO_2RR performances of different catalysts in MEA-SSE electrolyzer.
Supplementary Table 4. Comparison of eCO_2RR performances of different catalysts with air as feedstock in flow cell.

Materials and Methods

All reagents were commercially available and used without further purification. Powder X-ray diffraction (PXRD) patterns were recorded on a Bruker D8 Advance diffractometer (Cu K_α). Scanning electron microscope (SEM) images were obtained by a SU8010 system. High resolution transmission electron microscope (HR-TEM) images were obtained by an FEI Tecnai G^2 F30. Elemental analyses (EA) were conducted using an Elementar Vario EL analyzer. Inductively coupled plasma atomic emission spectrometry (ICP-AES) tests were performed on an iCAP6500Duo (Thermo Fisher). X-ray photoelectron spectroscopy (XPS) measurements were performed on an ESCALAB 250 spectrometer (Thermo-VG Scientific). Nuclear magnetic resonance (NMR) data were collected on a Bruker AVANCE IIIT 400HD.

Synthesis of MAF-4

1.68 g of $Zn(NO_3)_2 \cdot 6H_2O$ was dissolved in 80 mL of methanol and stirred for 20 min. In a separate vial, 3.7 g of 2-methylimidazole was dissolved in 80 mL of methanol under vigorous stirring. The second solution was then added dropwise to the first solution while continuously stirring to form a white suspension. The resulting mixture was stirred at room temperature for 24 h. Finally, the products were collected by filtration, washed three times with methanol, and dried under vacuum at 60 ℃.

Synthesis of CALF-20

6.60 g of ZnC_2O_4, 5.00 g of 1,2,4-triazole, and 66.0 mL of methanol were added into a 125 mL Teflon autoclave. The autoclave was then heated in a convection oven at 180 ℃ for 48 h. After cooling to room temperature, the products were collected by filtration, washed three times with ethanol, and dried under vacuum at 60 ℃.

Synthesis of HKUST-1

1.82 g of $Cu(NO_3)_2 \cdot 3H_2O$ and 0.875 g of trimesic acid was dissolved in 50 mL of methanol. The mixed solution was stirred at room temperature for 2 h. The obtained blue precipitate was collected by centrifugation, which was washed with ethanol for three times and then dried under vacuum at 60 ℃.

Synthesis of NH_2-MIL-53(Al)

0.724 g of $AlCl_3 \cdot 6H_2O$, 0.543 g of 2-aminoterephthalic acid (NH_2-H_2BDC) and 30 mL of distilled water were added into a 50 mL Teflon autoclave and sonicated for another 30 min. Then the autoclave was heated in a convection oven at 150 ℃ for 6 h. After cooling to room temperature, the obtained yellow precipitate was collected by centrifugation, which was washed with ethanol for three times and then dried under vacuum at 60 ℃.

Synthesis of KAUST-7

1.74 g of $Ni(NO_3)_2 \cdot 6H_2O$, 0.8 g of Nb_2O_5, 3.84 g of pyrazine, 2.6 mL of 48% HF_{aq} and 30 mL of distilled water were added into a 50 mL Teflon autoclave and sonicated for another 30 min. Then the autoclave was heated to 130 ℃ for 24 h. After cooling to room temperature, the obtained light bule precipitate was collected by centrifugation, which was washed with ethanol for three times and then dried under vacuum at 60 ℃.

Gas separation measurements

We constructed two gas channels that were separated by a MOF-MMM. Flue gas with a fixed volume ratio (15% CO_2, 81% N_2, 4% O_2, 500 ppm NO and 500 ppm SO_2) flowed through one channel at a rate of 50 sccm with the pressure of 2 bar controlled by a back pressure valve. The carrier gas (Ar) was flowed through the other channel at a rate of 30 sccm to a gas chromatography for gas sampling. The signals of CO_2, N_2 and O_2 were quantified by a thermal conductivity detector (TCD). Air separation measurements are similar to flue gas, except that the rate of air is 200 sccm instead of 50 sccm.

Determination of SO_2

The SO_2 concentration was measured by colorimetry using formaldehyde-pararosaniline hydrocloride method. Typically, 1.82 g of 1,2-diaminocyclohexane-*N*,*N*,*N'*,*N'*-tetraacetic acid and 0.39 g of NaOH were dissolved in 100 mL of H_2O, denoted as CDTA-2Na solution. Then 0.55 mL of 37% formaldehyde and 2 mL of CDTA-2Na solution were added into 97.5 mL of H_2O, denoted as solution A. 0.6 g of sulfamic acid and 0.24 g of NaOH were added into 100 mL of H_2O, denoted as solution B. 0.05 g of pararosaniline, 30 mL of 85% phosphoric acid and 12 mL of 37% hydrochloric acid were added into 58 mL of H_2O, denoted as solution C. By continuously introducing flue gas passing through MMM into 100 mL of solution A for 10 minutes, then mixing 10 mL of solution A, 0.5 mL of solution B, 0.5 mL of solution C and 0.5mL of 1.5 M NaOH aqueous solution and keeping the mixture in a dark box at room temperature for 1 h, the absorbance curve with the maximum absorbance at 560 nm was collected by an ultraviolet–visible spectrophotometer. The concentration–absorbance curves were calibrated by using standard SO_2 for a series of concentrations, as shown in Supplementary Figure 21.

Determination of NO

The NO concentration was measured by colorimetry using the cerium sulfate titration method [1]. Typically, 5 g of sulfanilic acid (SA) and 50 mg of *N*-(-1-naphthyl)ethylenediamine dihydrochloride (NED) were dissolved in a mixed solution of 50 mL of acetic acid and 950 mL of H_2O, denoted as SA-NED solution. By continuously introducing flue gas passing through MMM into 100 mL of SA-NED solution for 10 minutes, the sulfonamide reacts with the NO to form a diazonium salt and then further reacts with the amine to form an azo dye (magenta). After keeping the mixture in a dark box at room temperature for 15 min, the absorbance curve with the maximum absorbance at 530.5 nm was collected by an ultraviolet–visible spectrophotometer. The concentration–absorbance curves were calibrated by using standard NO for a series of concentrations, as shown in Supplementary Figure 22.

Preparation of working electrode

Firstly, 20 mg of poly[4,5-difluoro-2,2-bis(trifluoromethyl)-1,3-dioxole-co-tetrafluoroethylene] (PT) was dispersed into the 1 mL of FC-770 electronic liquid as the polymer solution. Then, 10 mg of Bi NPs was added into a mixed solution of FC-770 electronic liquid (950 μL) and polymer solution (50 μL), followed by sonication for 30 min to obtain a uniform dispersed ink. This ink (50 μL) was dropped onto hydrophobic carbon paper (Sigracet 29 BC) of a gas diffusion layer electrode with an area of 1 cm^2, affording a high surface area and enabling rapid mass transport of gas-phase CO_2, and then dried at room temperature. The average mass loading was 0.5 mg cm^{-2}.

Determination of HCOOH concentration

The concentration of HCOOH in the electrolyte was measured by ^{1}H NMR spectroscopy. A mixed solution of electrolyte (500 μL) after electrocatalysis in the cathode chamber, deuterated water (100 μL) and DMSO (5000 ppm, 100 μL) were quantified by ^{1}H NMR spectroscopy with solvent (H_2O) suppression. The concentration–integral area curves were calibrated by using standard HCOOH solutions for a series of concentrations, which contained the salt solution as same as electrolyte (0.05 M H_2SO_4 + 0.5 M K_2SO_4), as shown in Supplementary Figure 4. The FE_{HCOOH} was calculated using the following formula:

$$\mathrm{FE_{HCOOH}} = \frac{2 \times F \times C_{\mathrm{HCOOH}} \times V}{46.03 \times Q}$$

where C_{HCOOH} is the measured concentration of HCOOH (g L^{-1}), V is the total volume of electrolyte (mL), F is Faraday constant (96485 C mol^{-1}) and Q is the quantity charge (C).

Determination of NH_3

The amount of NH_3 in the solution was determined by colorimetry using the indophenol blue colorimetric method [2]. Typically, 2 mL of 1 M NaOH solution containing 5 wt% salicylic acid and 5 wt% sodium citrate, NaClO (0.05 M, 1 mL) and sodium nitroferricyanide (1 wt%, 0.2 mL) were added in turn to the mixed solution of NH_3-containing solution (0.4 mL) and KOH (1 M, 1.6 mL) and mixed vigorously. After leaving the mixture to stand at room temperature in the dark for 2 h, the absorbance curve with the maximum absorbance at 658.5 nm was collected by an ultraviolet–visible spectrophotometer. The concentration–absorbance curves were calibrated by using standard ammonium chloride solutions for a series of concentrations, which contained the salt solution as same as electrolyte (0.05 M H_2SO_4 + 0.5 M K_2SO_4), as shown in Supplementary Figure 6. The FE of NH_3 was calculated using the following formula:

$$\mathrm{FE_{NH_3}} = \frac{5 \times F \times C_{\mathrm{NH_3}} \times V}{17.03 \times Q}$$

where $C_{\mathrm{NH_3}}$ is the measured concentration of NH_3 (mg L^{-1}), V is the total volume of electrolyte (mL), F is Faraday constant (96485 C mol^{-1}) and Q is the quantity charge (C).

Determination of H_2O_2

The H_2O_2 concentration was measured by colorimetry using the cerium sulfate titration method [3]. Typically, 0.1 mL of solution with H_2O_2 product was mixed with 5 mL of 0.4 mM $Ce(SO_4)_2$ aqueous solution with 0.5 M H_2SO_4. After leaving the mixture to stand at room temperature in the dark for 6 min, the absorbance curve with the maximum absorbance at 319 nm was collected by an ultraviolet–visible spectrophotometer. The concentration–absorbance curves were calibrated by using standard ammonium chloride solutions for a series of concentrations, which contained the salt solution as same as electrolyte (0.05 M H_2SO_4 + 0.5 M K_2SO_4), as shown in Supplementary Figure 7. The FE of H_2O_2 was calculated using the following formula:

$$\mathrm{FE_{H_2O_2}} = \frac{2 \times F \times C_{\mathrm{H_2O_2}} \times V}{34.02 \times Q}$$

where $C_{\mathrm{H_2O_2}}$ is the measured concentration of H_2O_2 (mg L^{-1}), V is the total volume of electrolyte (mL), F is Faraday constant (96485 C mol^{-1}) and Q is the quantity charge (C).

Techno-economic analysis

Preliminary estimation of the production cost for the electrosynthesis of HCOOH using a 1 cm^2 flow cell. We only calculated the costs of energy and feedstock input and no other costs associated with practical production or infrastructure were included. The price of electricity is 3 cents/kWh and the industrial CO_2 price is \$0.03/kg (DOI: 10.1126/science.aav3506).

Pure CO_2 condition: Operation condition: 4.5 V; 1.04 A; Average HCOOH Faradaic efficiency: 82.8%; Production rate: 16.06 mmol cm^{-2} h^{-1} (0.74 g h^{-1}); Operation time: 1 hour. Generated HCOOH: 0.74 g. Consumed CO_2: 0.71 g. Electricity cost: 3 cents/kWh $\times$ 4.5 V $\times$ 1.04 A $\times$ 1 h = 0.0140 cents. CO_2 cost: \$0.03/kg $\times$ 0.71 g = 0.002 cents. Total cost: 0.0160 cents.

Flow gas condition: Operation condition: 4.5 V; 1.01 A; Average HCOOH Faradaic efficiency: 83.4%; Production rate: 15.71 mmol cm^{-2} h^{-1} (0.72 g h^{-1}); Operation time: 1 hours. Generated HCOOH: 0.72 g. Consumed CO_2: 0.69 g. Electricity cost: 3 cents/kWh $\times$ 4.5V $\times$ 1.01 A $\times$ 1 h = 0.0136 cents. CO_2 cost: 0 cents. Total cost: 0.0136 cents.

Therefore, compared with the condition of pure CO_2, the use of flue gas can reduce the cost by 15%.

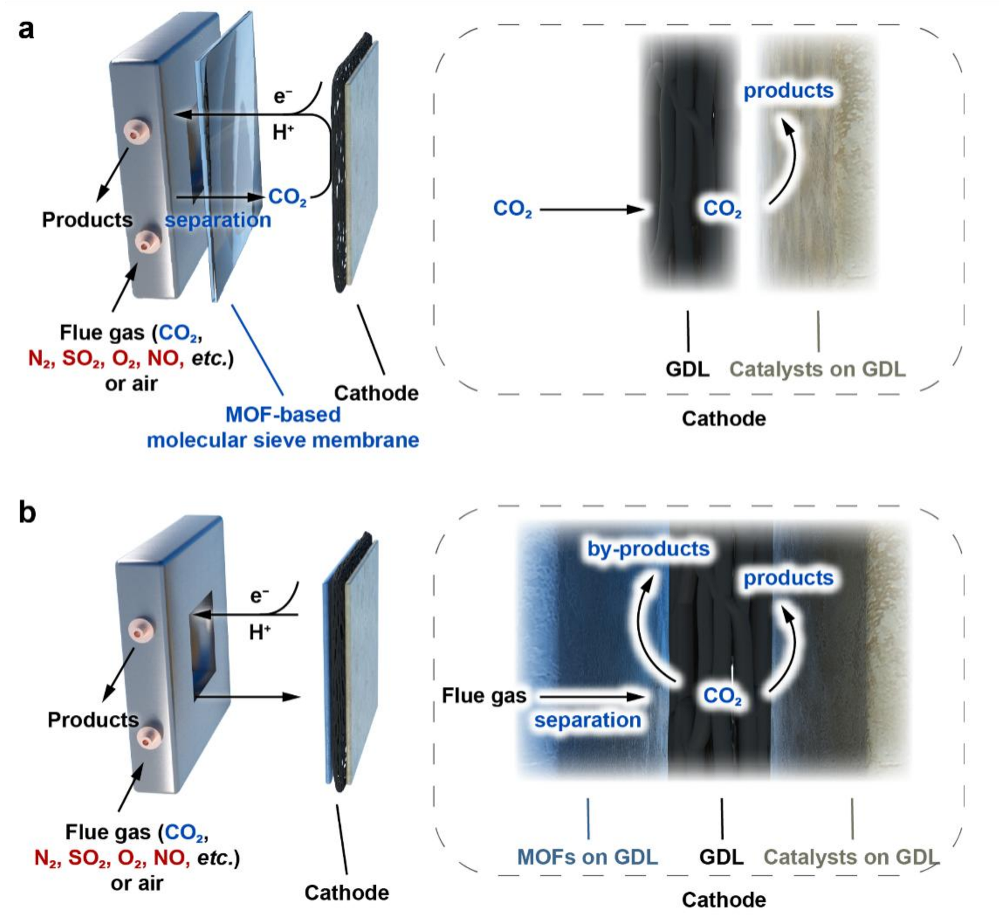

Supplementary Figure 1. Comparison between the flow cells. Comparison between the flow cell embedded with a self-supporting MOF-based molecular sieve membrane (a) and the reported flow cell featuring a multifunctional working cathode (b) composed of MOFs, GDL and catalyst highlights distinct advantages.

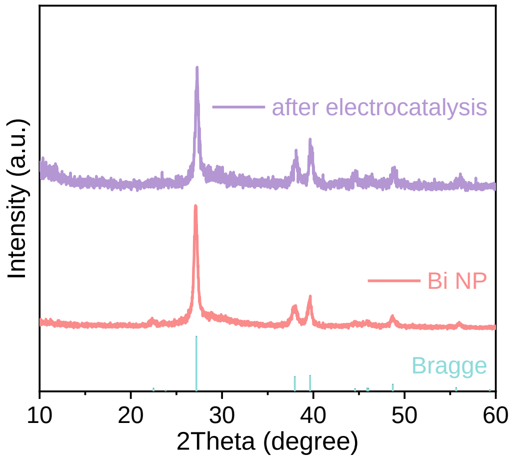

Supplementary Figure 2. PXRD characterization of Bi NPs. PXRD patterns of Bi NPs before and after electrocatalysis.

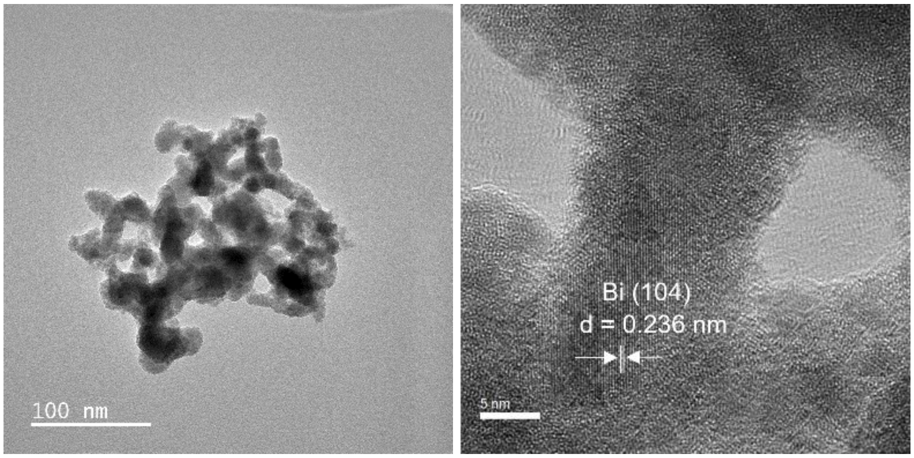

Supplementary Figure 3. HR-TEM images of Bi NPs.

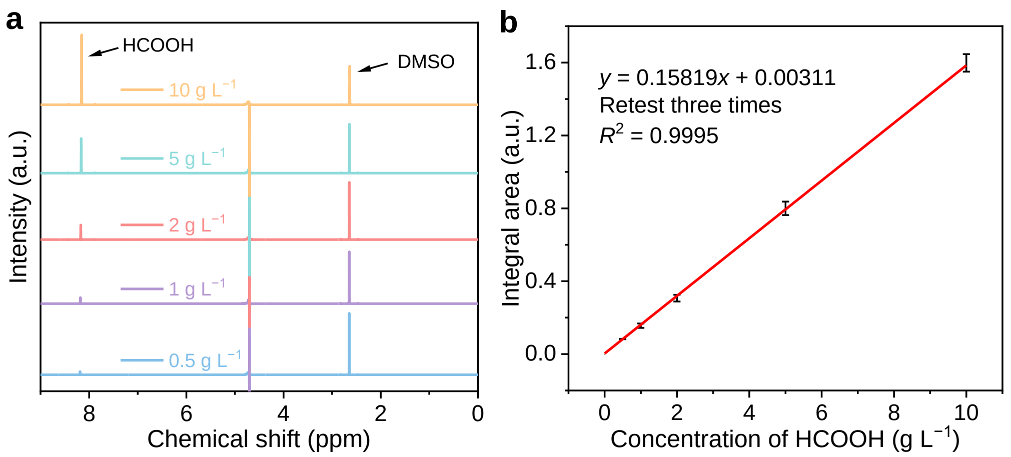

Supplementary Figure 4. Measurements of the HCOOH concentrations by ^{1}H NMR spectroscopy. (a) ^{1}H NMR spectra of a series of HCOOH standard solutions (0, 0.5, 1, 2, 5 and 10 g L^{-1}). (b) The calibration curve for quantification of HCOOH indicates good linear relation of integral area with HCOOH concentration (y = 0.15819x + 0.00311, R^2 = 0.9995).

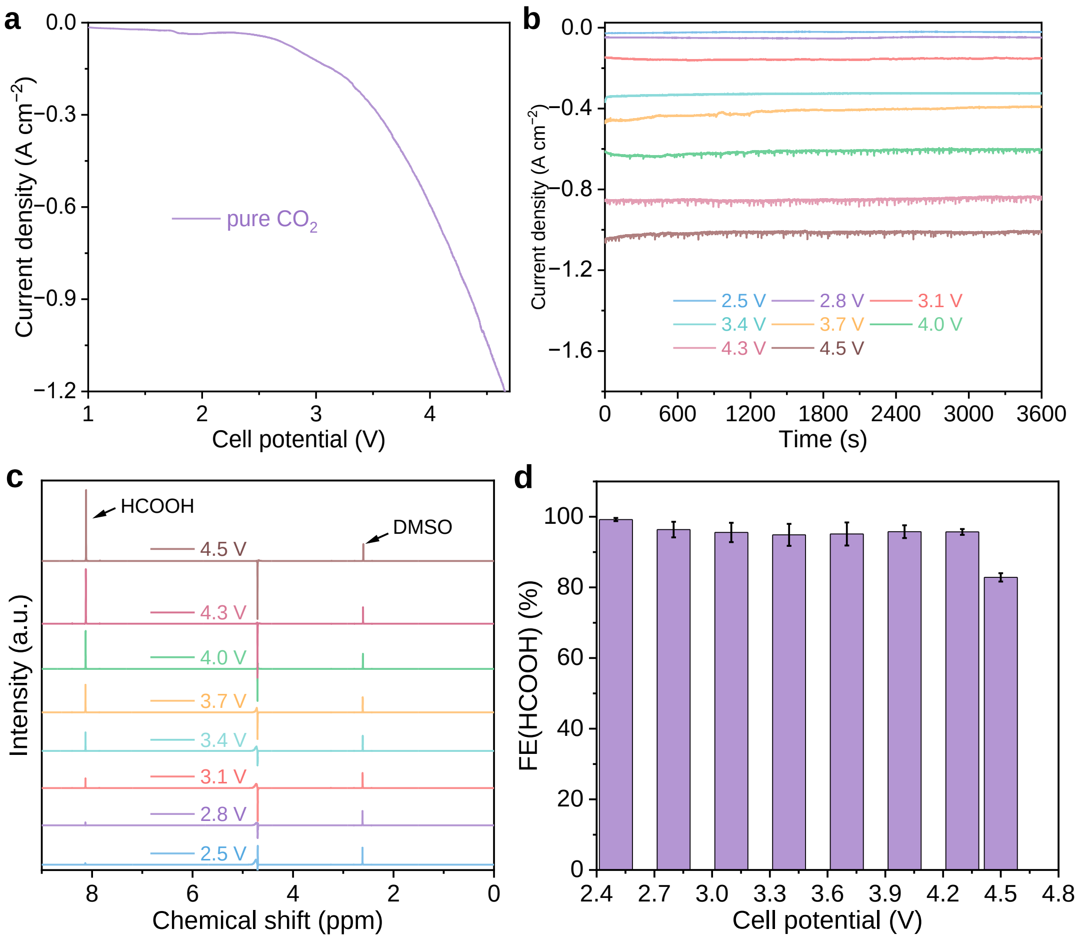

Supplementary Figure 5. Performance of eCO_2RR with pure CO_2 as feedstock by Bi NPs. (a) LSV curve in pure CO_2 gas. (b) *i*-*t* curves at different potentials. (c) ^1H NMR measurements and (d) FE_{HCOOH} with pure CO_2 as feedstock at the potentials of 2.5 V, 2.8 V, 3.1 V, 3.4 V, 3.7 V, 4.0 V, 4.3 V and 4.5 V, respectively.

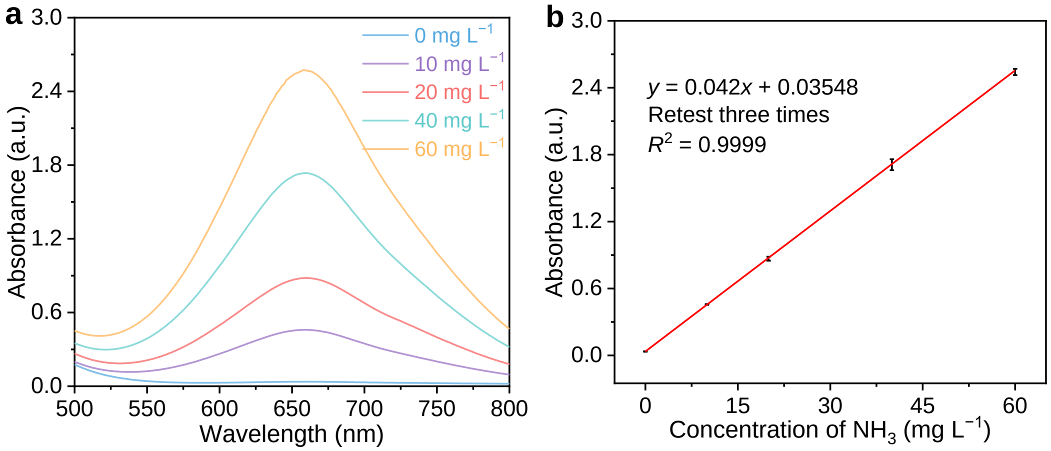

Supplementary Figure 6. UV-vis measurements of the concentrations of NH_3. (a) UV-vis spectra of a series of NH_3 standard solutions (0, 10, 20, 40 and 60 mg L^{-1}). (b) The calibration curve for quantification of NH_3 indicates good linear relation of absorbance with NH_3 concentration (y = 0.042x + 0.03548, R^2 = 0.9999). Each data point on the calibration curve was obtained by averaging the results of three independent measurements.

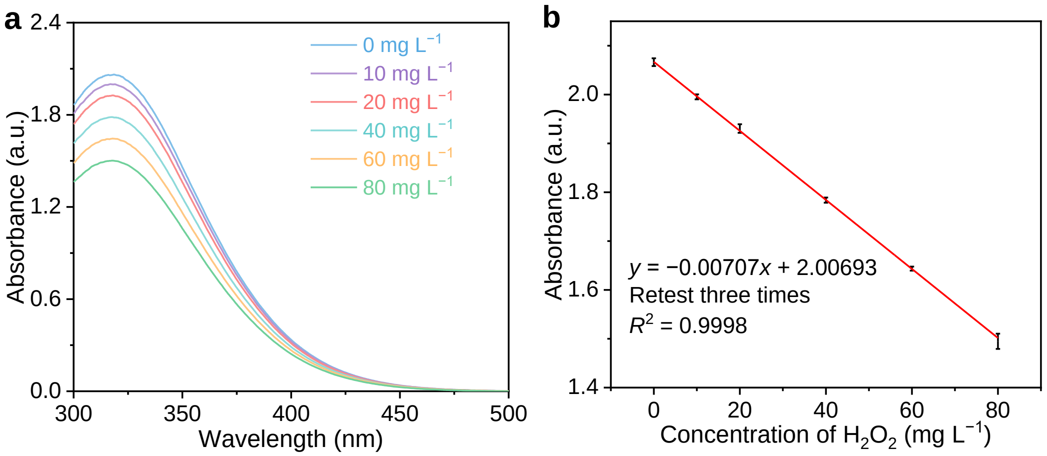

Supplementary Figure 7. UV-vis measurements of the concentrations of H_2O_2. (a) UV-vis spectra of a series of H_2O_2 standard solutions (0, 10, 20, 40, 60 and 80 mg L^{-1}). (b) The calibration curve for quantification of H_2O_2 indicates good linear relation of absorbance with H_2O_2 concentration ($y = -0.00707x + 2.00693$, $R^2 = 0.9998$). Each data point on the calibration curve was obtained by averaging the results of three independent measurements.

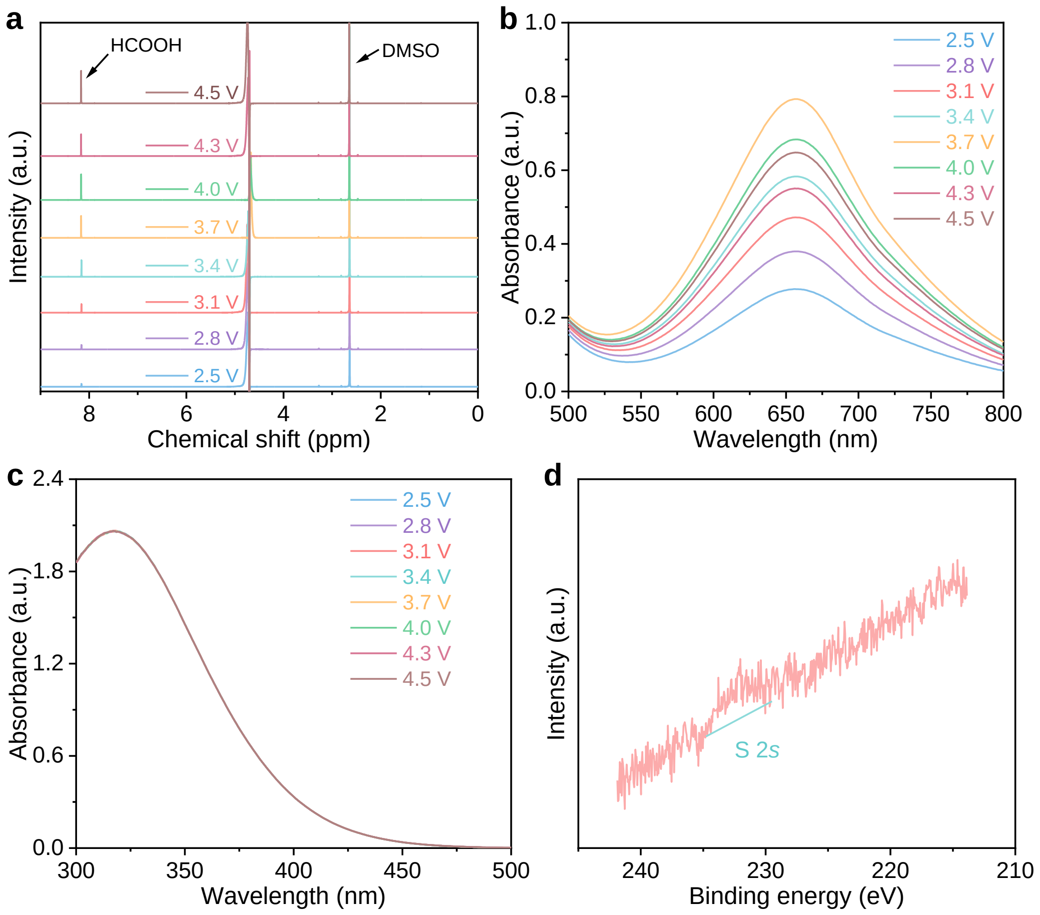

Supplementary Figure 8. ^{1}H NMR and UV-vis measurements of products by Bi/GDL with flue gas as feedstock. (a) ^{1}H NMR measurements of HCOOH and UV-vis measurements of NH_3 (b) and H_2O_2 (c) with flue gas as feedstock at the potentials of 2.5 V, 2.8 V, 3.1 V, 3.4 V, 3.7 V, 4.0 V, 4.3 V and 4.5 V, respectively. (d) The XPS spectra of S 2*s* of Bi NPs after electrocatalysis with flue gas as feedstock.

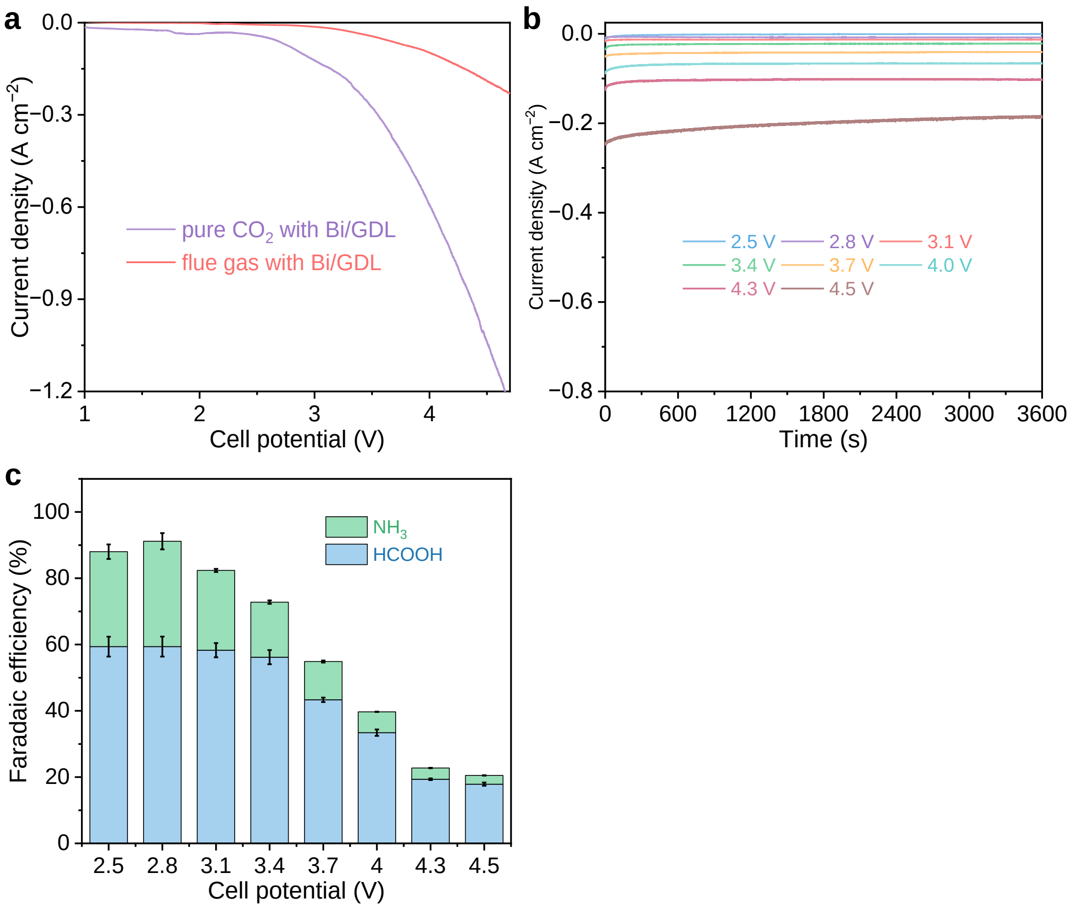

Supplementary Figure 9. Performance of eCO_2RR by Bi/GDL with flue gas as feedstock. (a) LSV curves, (b) *i*-*t* curves and (c) FEs of HCOOH and NH_3.

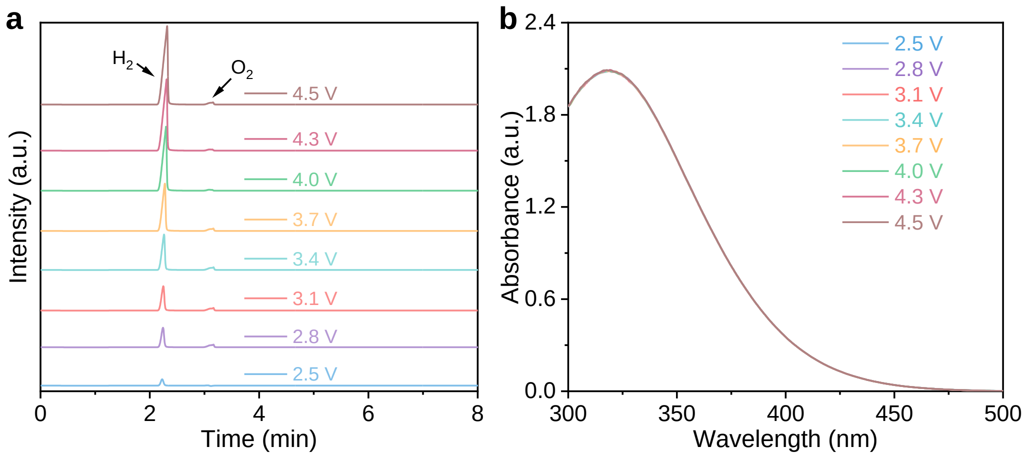

Supplementary Figure 10. GC and UV-vis measurements of products by Bi/GDL with O_2/N_2 (20/80) as feedstock. GC profile of H_2 and (b) UV-vis measurements of H_2O_2 at the potentials of 2.5 V, 2.8 V, 3.1 V, 3.4 V, 3.7 V, 4.0 V, 4.3 V and 4.5 V, respectively.

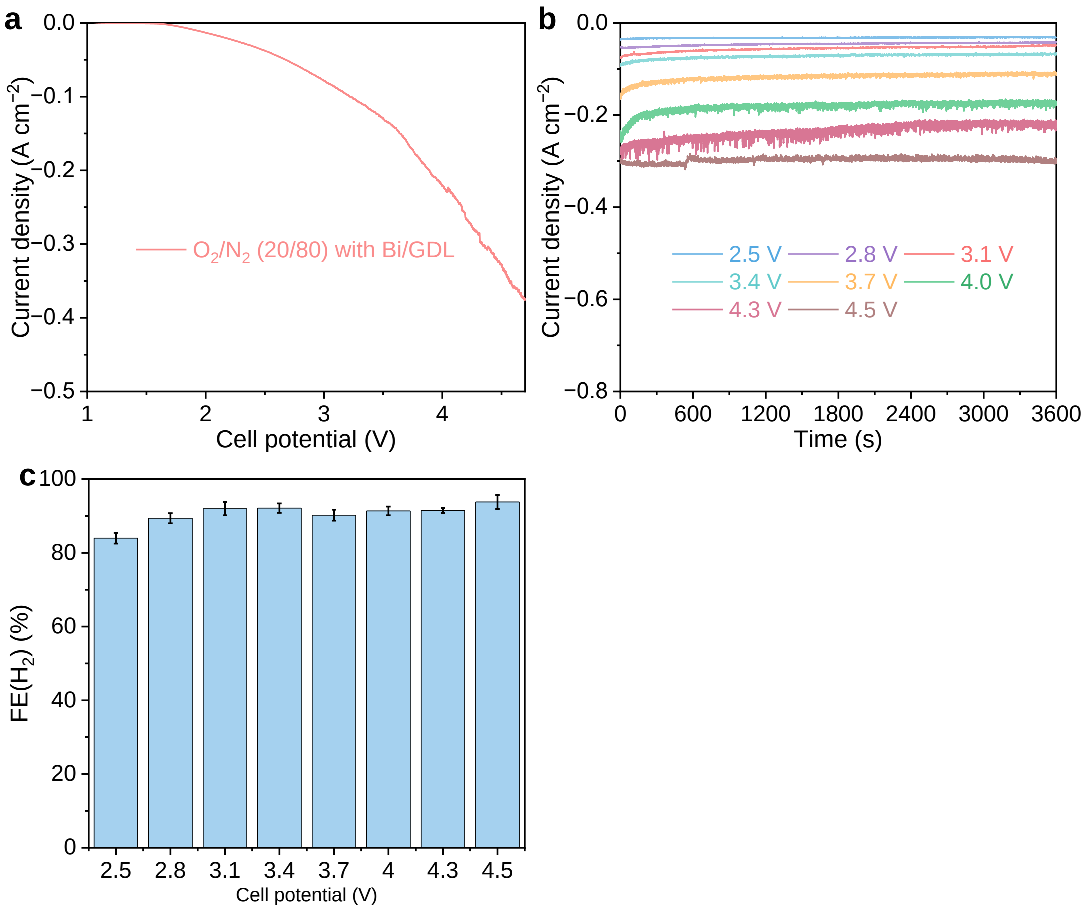

Supplementary Figure 11. Performance of eCO_2RR by Bi/GDL with O_2/N_2 (20/80) as feedstock. (a) LSV curve, (b) *i*-*t* curves and (c) FE of H_2.

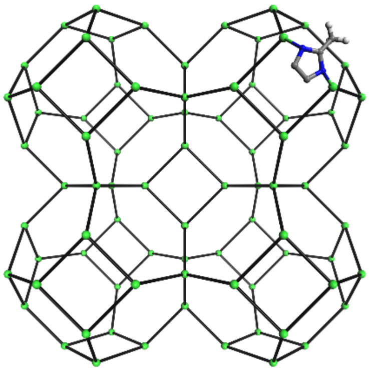

Supplementary Figure 12. Schematic diagram of **MAF-4**. Green nodes representing Zn^{2+} ions.

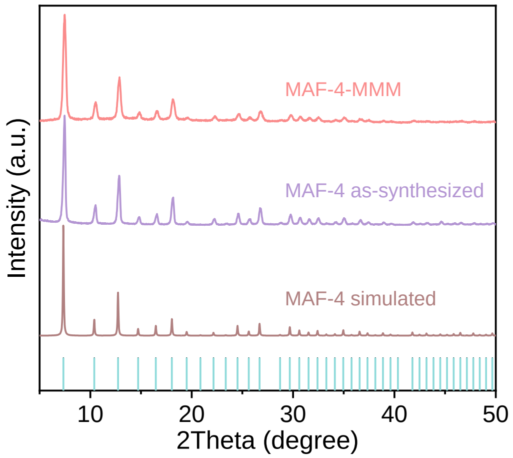

Supplementary Figure 13. PXRD characterization of **MAF-4**. PXRD patterns of as-synthesized and simulated **MAF-4**, as well as **MAF-4**-MMM.

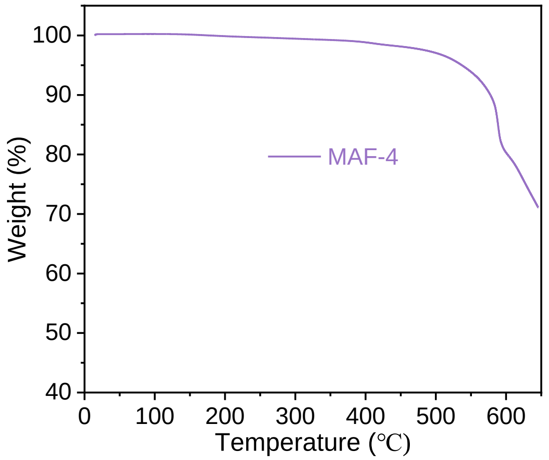

Supplementary Figure 14. Thermogravimetric curve characterization of **MAF-4**. It can be seen that the framework of **MAF-4** remains thermostable when heated to ca. 500 °C.

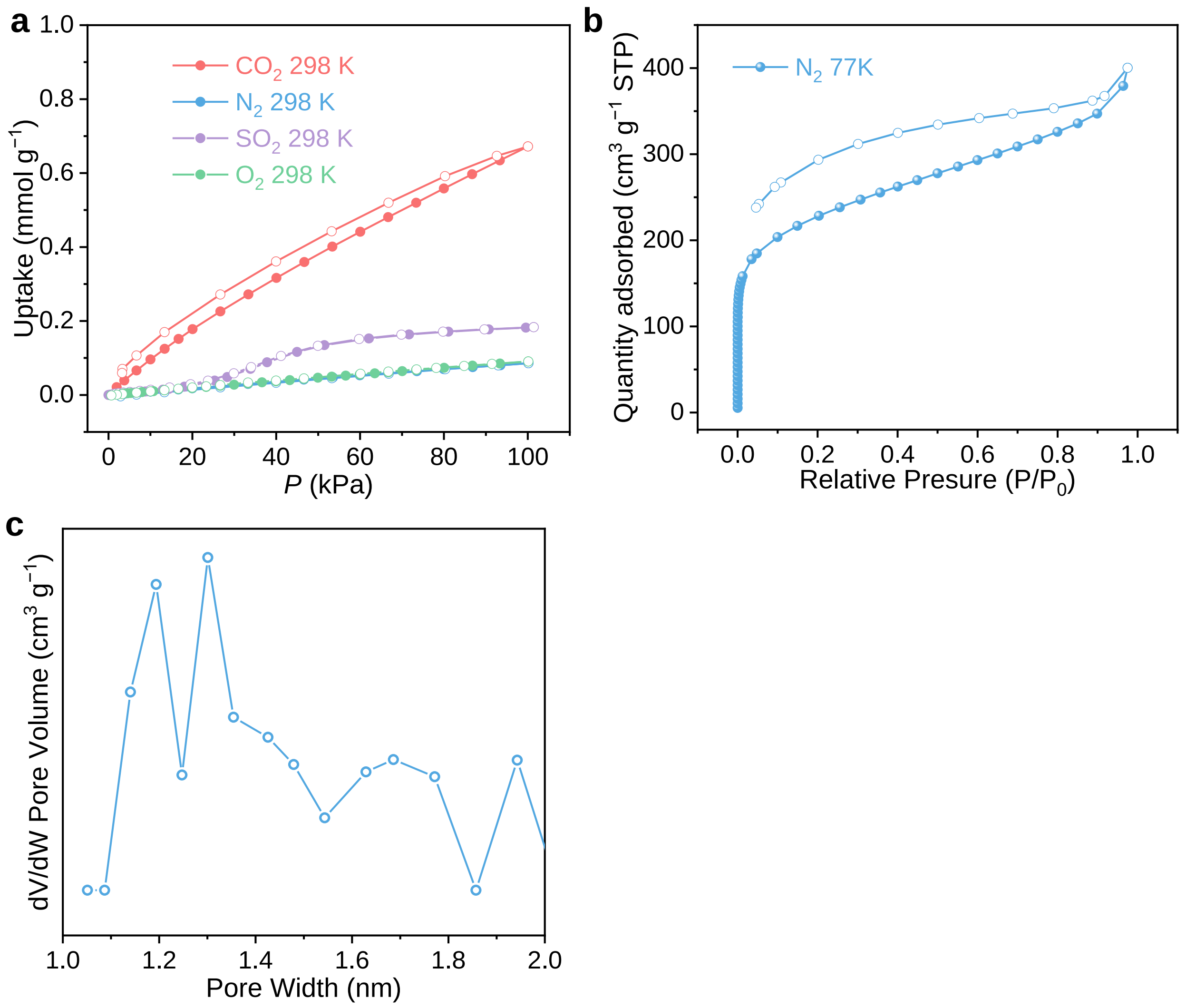

Supplementary Figure 15. CO_2, N_2, SO_2 and O_2 sorption isotherms of **MAF-4** at 298 K. (a) CO_2, N_2, SO_2 and O_2 adsorption (solid) and desorption (open) isotherms of **MAF-4** measured at 298 K. (b) N_2 adsorption (solid) and desorption (open) isotherms of PIM-1 measured at 77 K. (c) Pore size distribution of PIM-1.

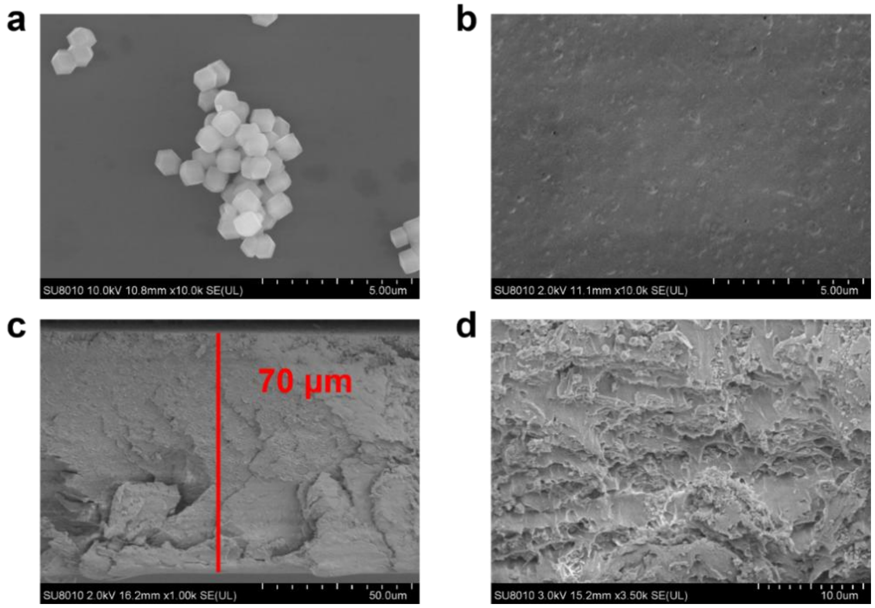

Supplementary Figure 16. SEM images of **MAF-4** and **MAF-4**-MMM. (a) SEM image of **MAF-4**. (b) SEM image of the surface of **MAF-4**-MMM. (c, d) SEM images of the cross section of **MAF-4**-MMM at different resolutions.

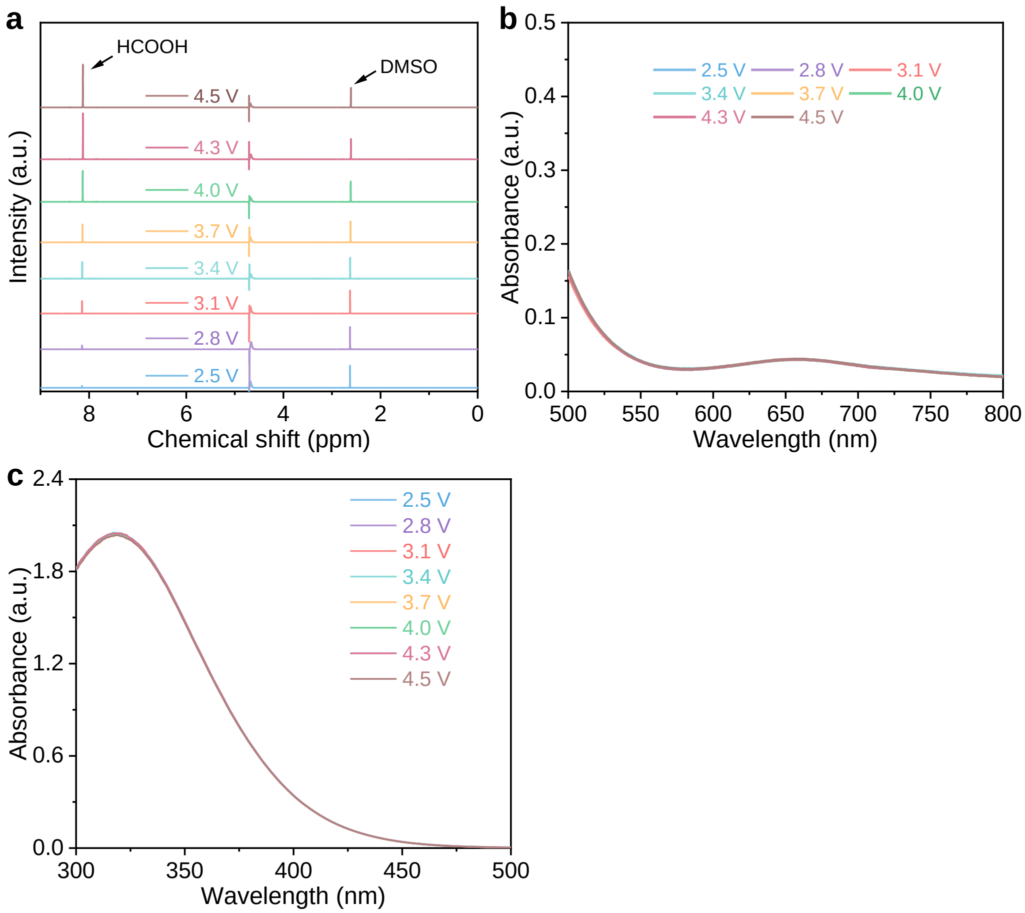

Supplementary Figure 17. ^{1}H NMR and UV-vis measurements of products by Bi/GDL with flue gas as feedstock in flow cell with **MAF-4**-MMM. (a) ^{1}H NMR measurements of HCOOH and UV-vis measurements of (b) NH_3 and (c) H_2O_2 with flue gas as feedstock at the potentials of 2.5 V, 2.8 V, 3.1 V, 3.4 V, 3.7 V, 4.0 V, 4.3 V and 4.5 V, respectively.

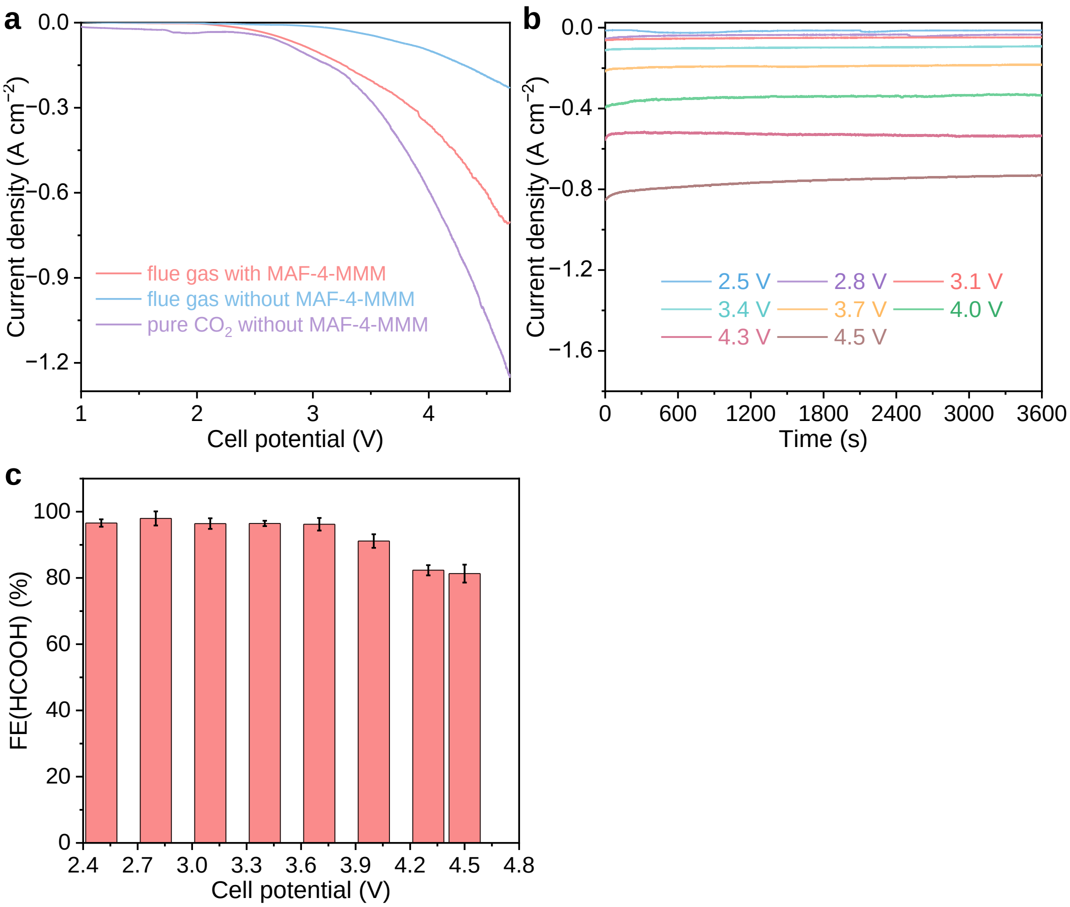

Supplementary Figure 18. Performance of eCO_2RR by Bi/GDL with flue gas as feedstock in flow cell with **MAF-4**-MMM. (a) LSV curves, (b) *i*-*t* curves and (c) FE of HCOOH.

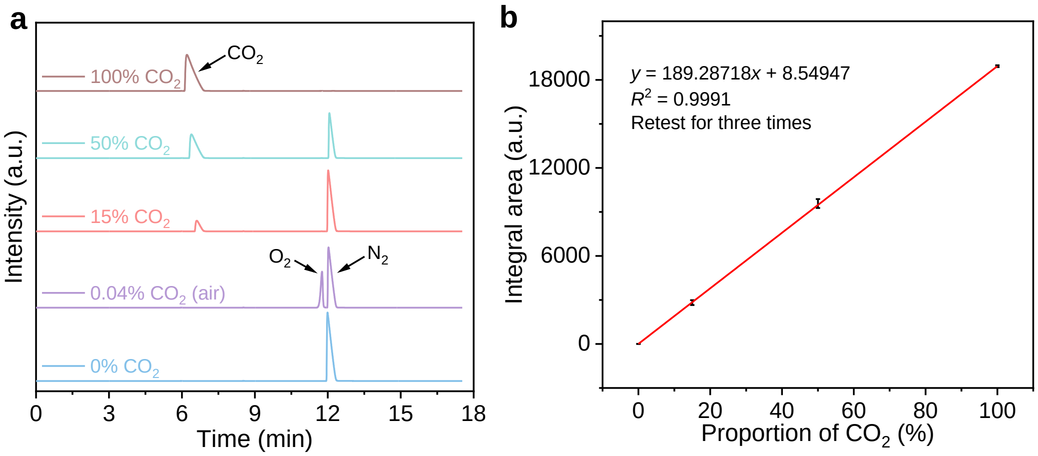

Supplementary Figure 19. GC profiles of different concentrations of CO_2. (a) GC profiles of a series of CO_2 standard mixed gas (0%, 0.04%, 15%, 50% and 100%). (b) The calibration curve for quantification of CO_2 indicates good linear relation of integral area with proportion of CO_2 ($y = 189.28718x + 8.54947$, $R^2 = 0.9991$). Each data point on the calibration curve was obtained by averaging the results of three independent measurements.

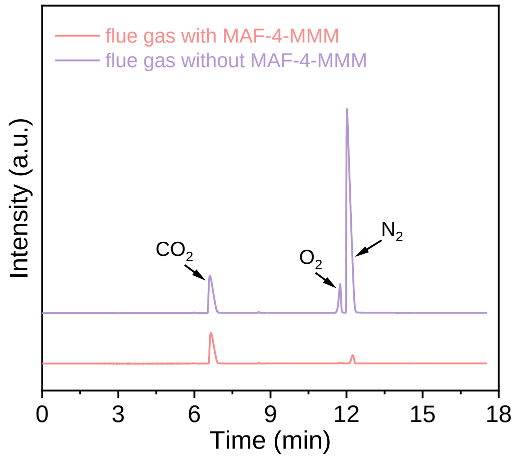

Supplementary Figure 20. GC profiles of CO_2. GC profiles of flue gas passing through **MAF-4**-MMM.

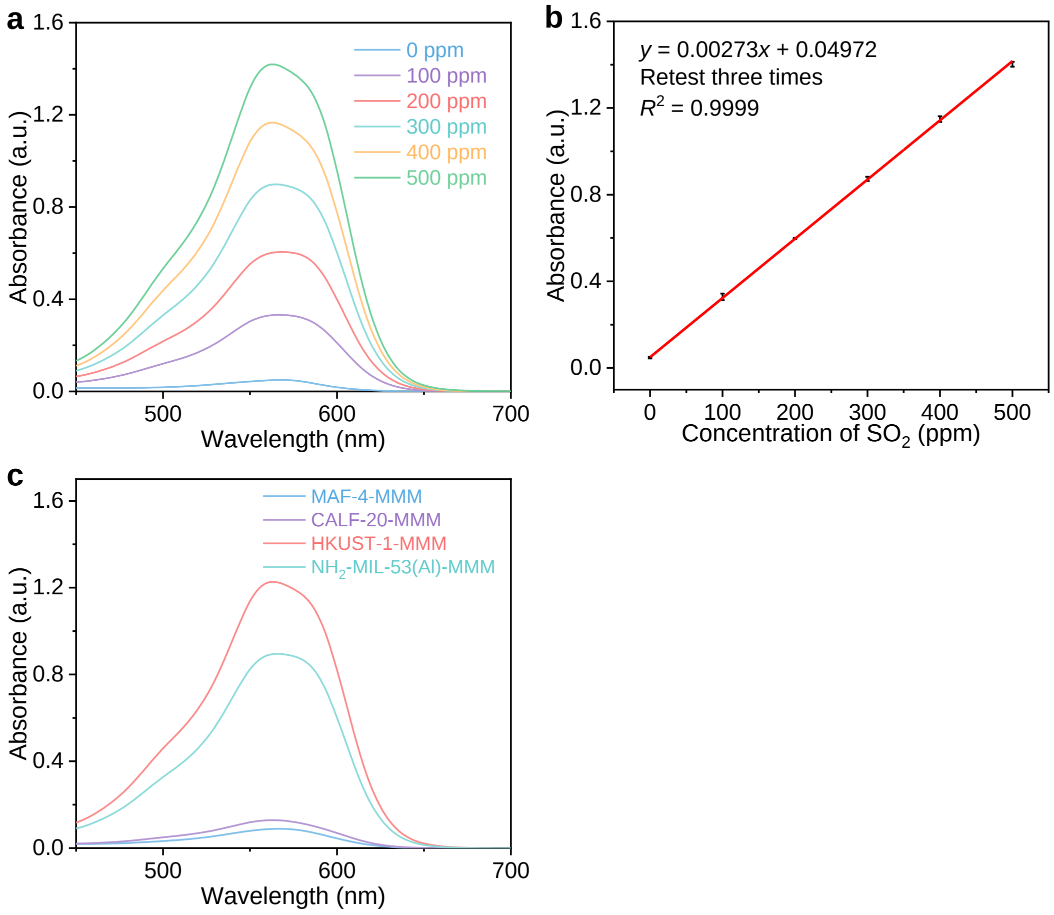

Supplementary Figure 21. UV-vis measurements of the concentrations of SO_2. (a) UV-vis spectra of a series of SO_2 standard gases (0, 100, 200, 300, 400 and 500 ppm). (b) The calibration curve for quantification of SO_2 indicates good linear relation of absorbance with SO_2 concentration ($y = 0.00273x + 0.04972$, $R^2 = 0.9999$). Each data point on the calibration curve was obtained by averaging the results of three independent measurements. (c) UV-vis measurements of SO_2 passing through **MAF-4**-MMM, **CALF-20**-MMM, **HKUST-1**-MMM and **NH_2-MIL-53(Al)**-MMM, respectively.

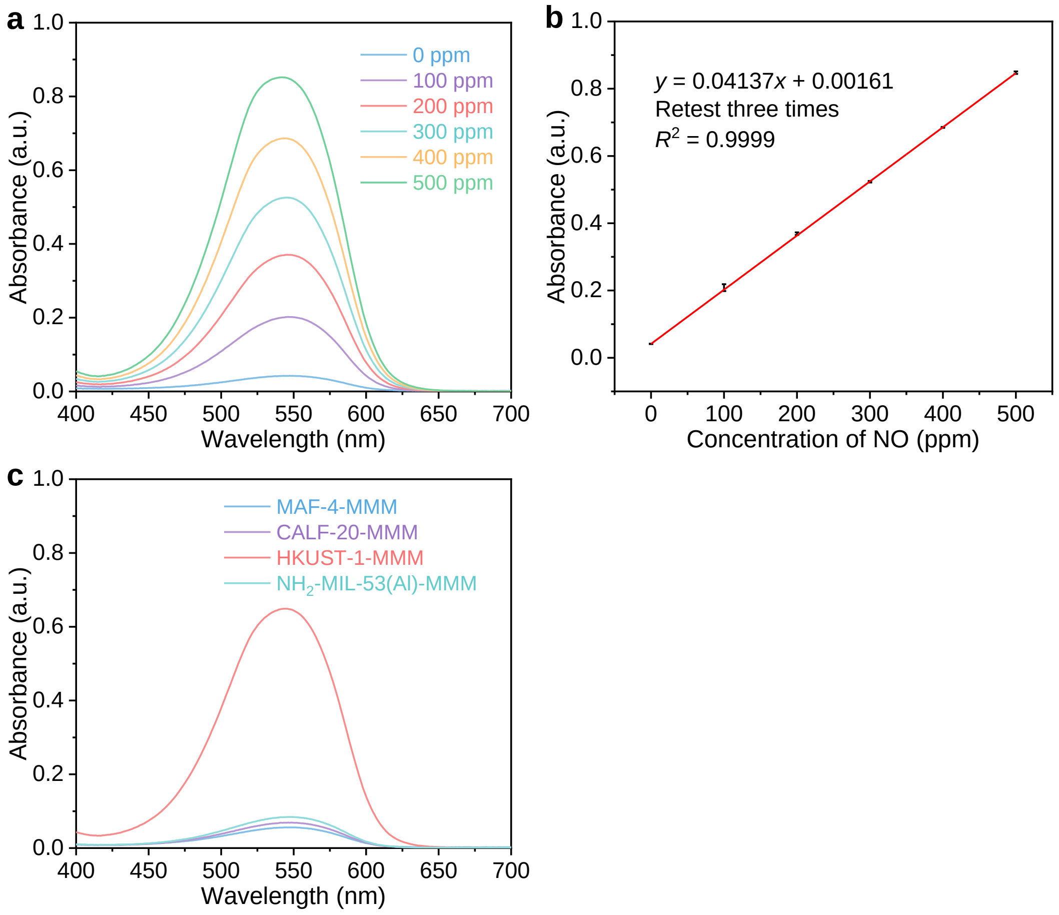

Supplementary Figure 22. UV-vis measurements of the concentrations of NO. (a) UV-vis spectra of a series of NO standard gases (0, 100, 200, 300, 400 and 500 ppm). (b) The calibration curve for quantification of NO indicates good linear relation of absorbance with NO concentration (y = 0.04137x + 0.00161, R^2 = 0.9999). Each data point on the calibration curve was obtained by averaging the results of three independent measurements. (c) UV-vis measurements of NO passing through **MAF-4**-MMM, **CALF-20**-MMM, **HKUST-1**-MMM and **NH_2-MIL-53(Al)**-MMM, respectively.

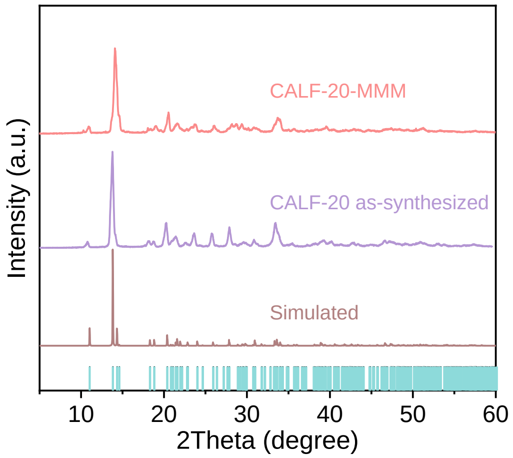

Supplementary Figure 23. PXRD characterization of **CALF-20**. PXRD patterns of as-synthesized and simulated **CALF-20**, as well as **CALF-20**-MMM.

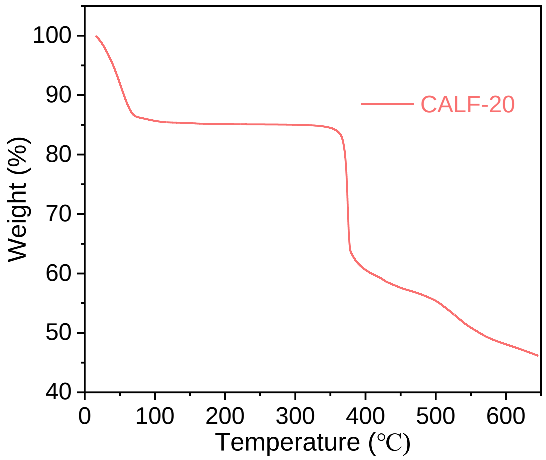

Supplementary Figure 24. TGA characterization of **CALF-20**. The first mass loss before 100 ℃ is due to the release of solvent molecules.

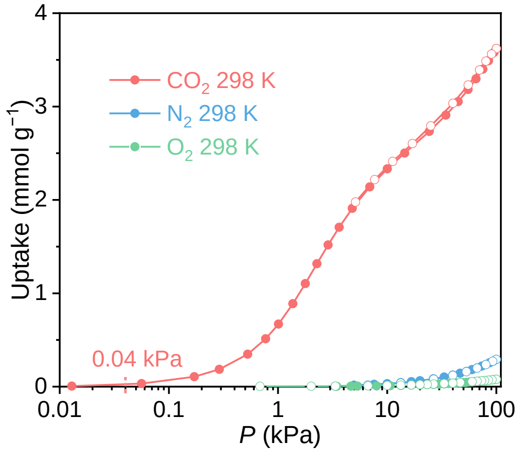

Supplementary Figure 25. CO_2, N_2 and O_2 sorption isotherms of **CALF-20** at 298 K. CO_2, N_2 and O_2 adsorption (solid) and desorption (open) isotherms of **CALF-20** measured at 298 K.

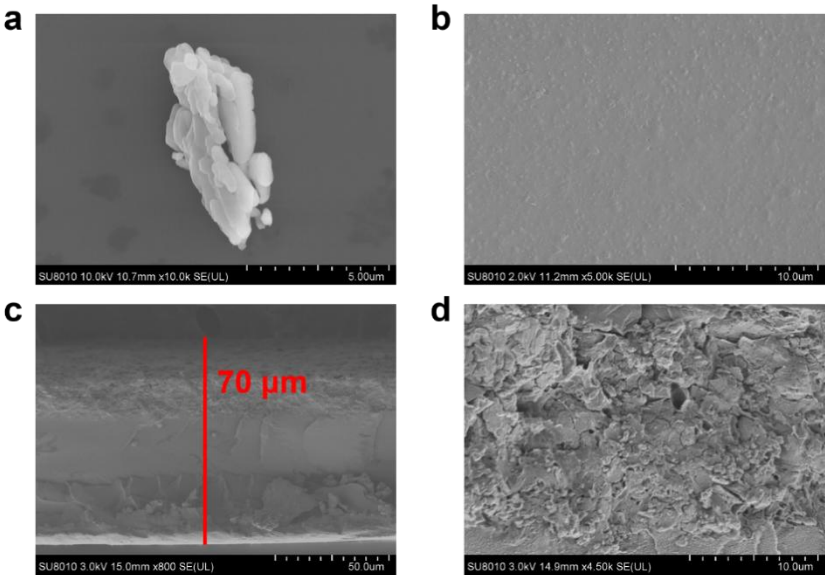

Supplementary Figure 26. SEM images of **CALF-20** and **CALF-20**-MMM. (a) SEM image of **CALF-20**. (b) SEM image of the surface of **CALF-20**-MMM. (c, d) SEM images of the cross section of **CALF-20**-MMM at different resolutions.

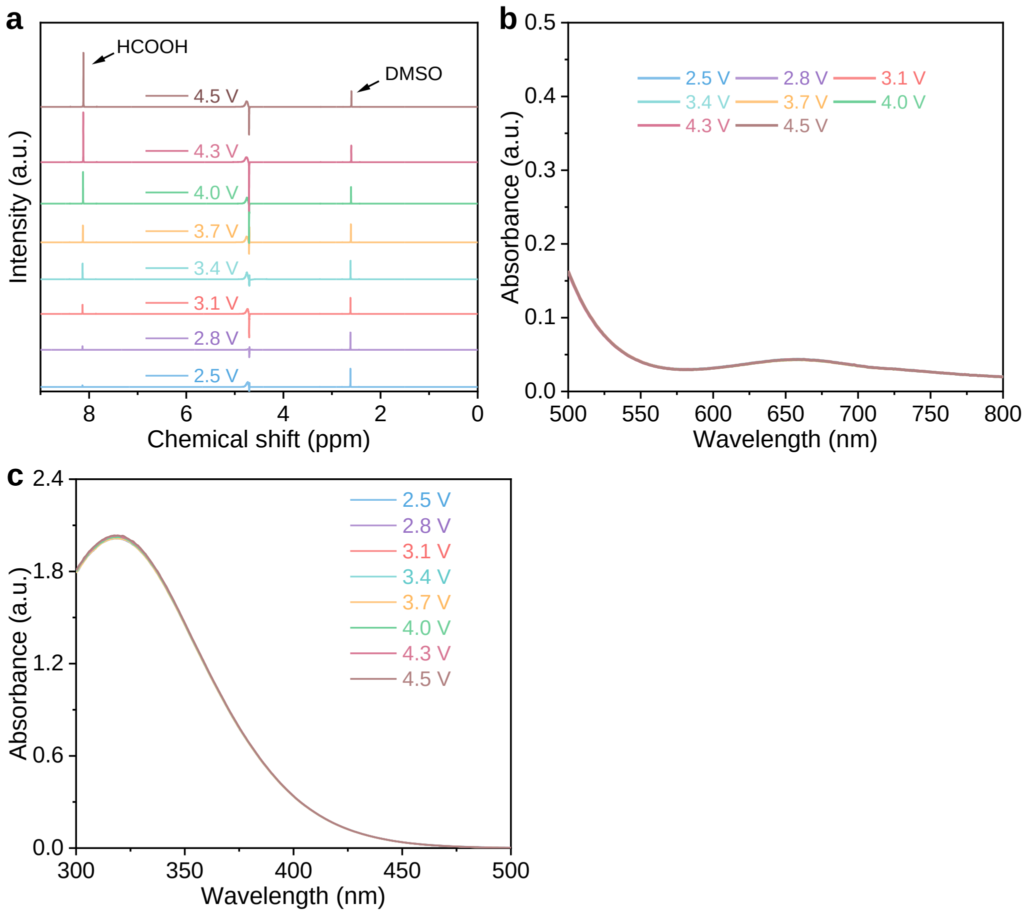

Supplementary Figure 27. ^{1}H NMR and UV-vis measurements of products by Bi/GDL with flue gas as feedstock in flow cell with **CALF-20**-MMM. (a) ^{1}H NMR measurements of HCOOH and (b) UV-vis measurements of (b) NH_3 and (c) H_2O_2 at the potentials of 2.5 V, 2.8 V, 3.1 V, 3.4 V, 3.7 V, 4.0 V, 4.3 V and 4.5 V, respectively.

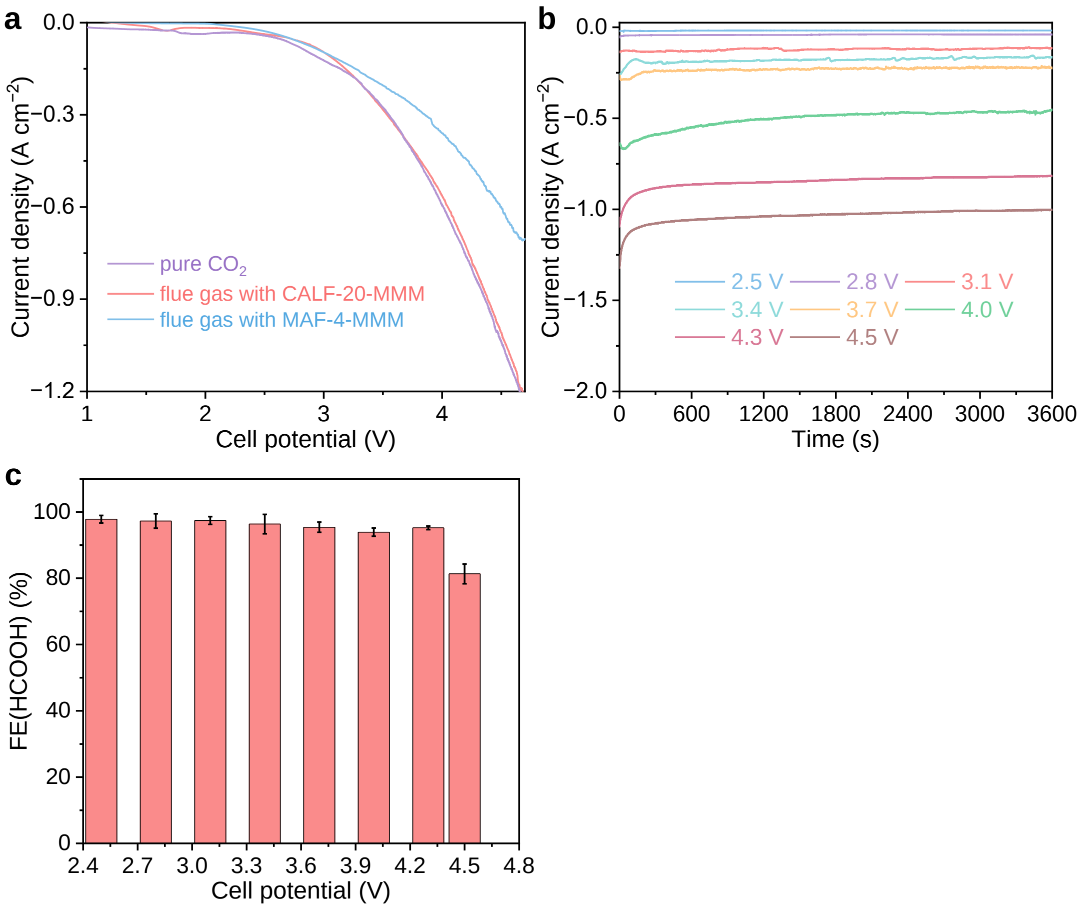

Supplementary Figure 28. Performance of eCO_2RR by Bi/GDL with flue gas as feedstock in flow cell with **CALF-20-**MMM. (a) LSV curves, (b) *i*-*t* curves and (c) FE_{HCOOH}.

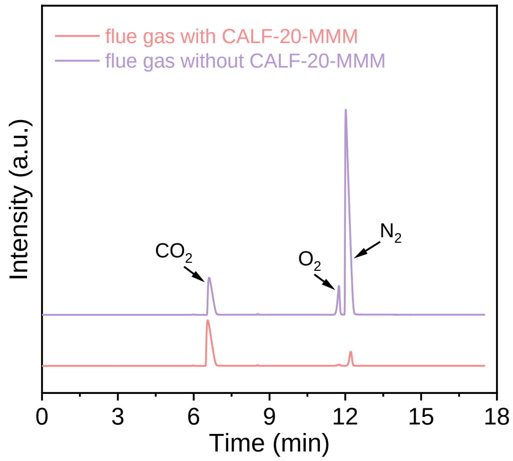

Supplementary Figure 29. GC profiles of CO_2. GC profiles of flue gas passing through **CALF-20**-MMM.

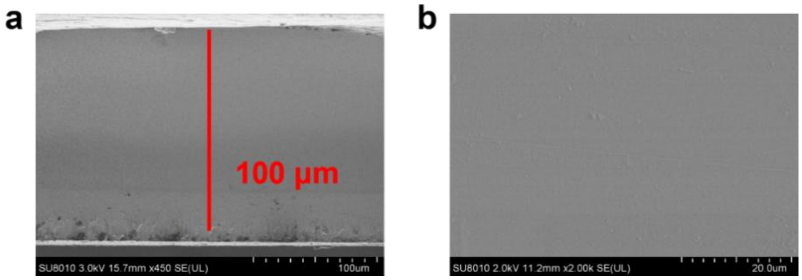

Supplementary Figure 30. SEM images of PIM-1 membrane. SEM images of the (a) cross section and (b) surface of PIM-1 membrane.

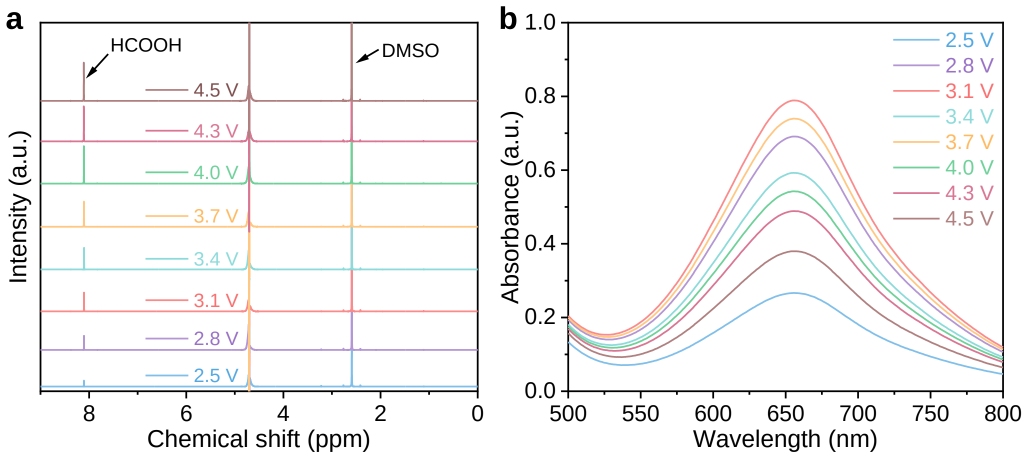

Supplementary Figure 31. ^{1}H NMR and UV-vis measurements of products by Bi/GDL with flue gas as feedstock in flow cell with PIM-1 membrane. (a) ^{1}H NMR measurements of HCOOH and (b) UV-vis measurements of NH_3 at the potentials of 2.5 V, 2.8 V, 3.1 V, 3.4 V, 3.7 V, 4.0 V, 4.3 V and 4.5 V, respectively.

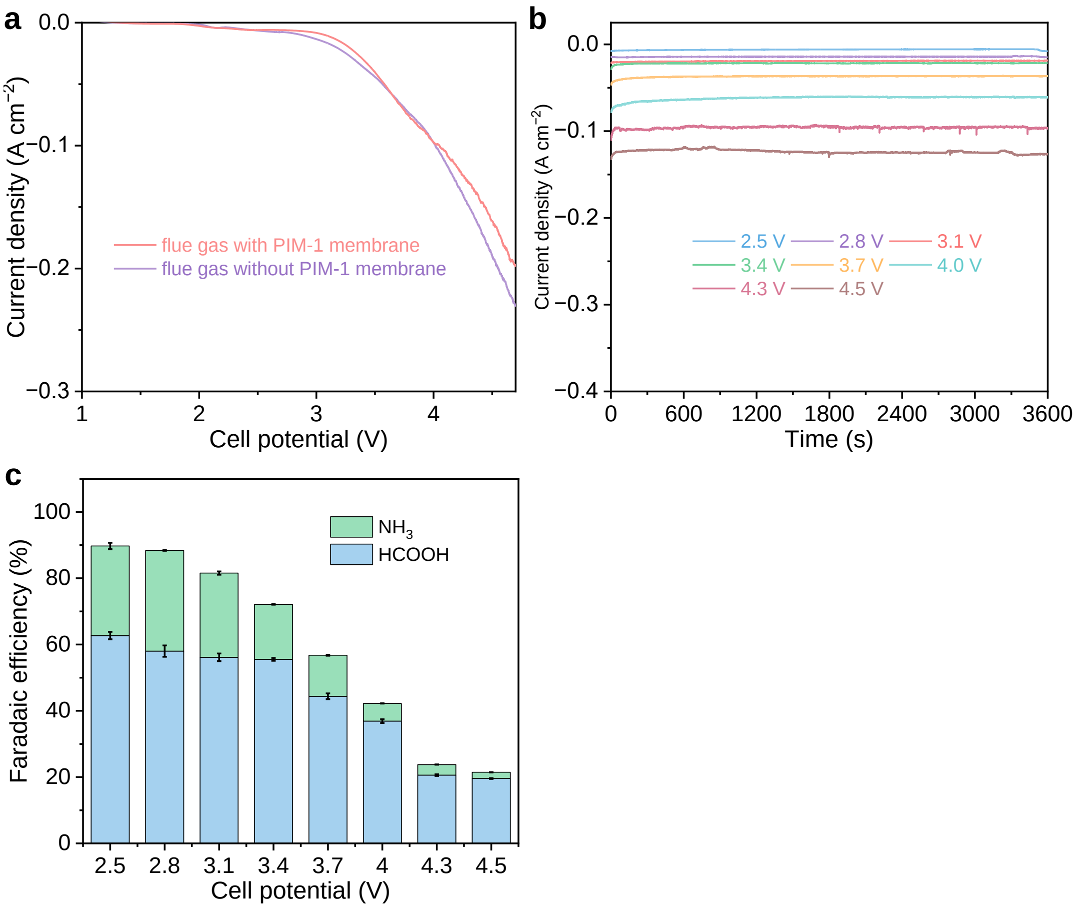

Supplementary Figure 32. Performance of eCO_2RR by Bi/GDL with flue gas as feedstock in flow cell with PIM-1 membrane. (a) LSV curve, (b) *i*-*t* curves and (c) FEs of HCOOH and NH_3.

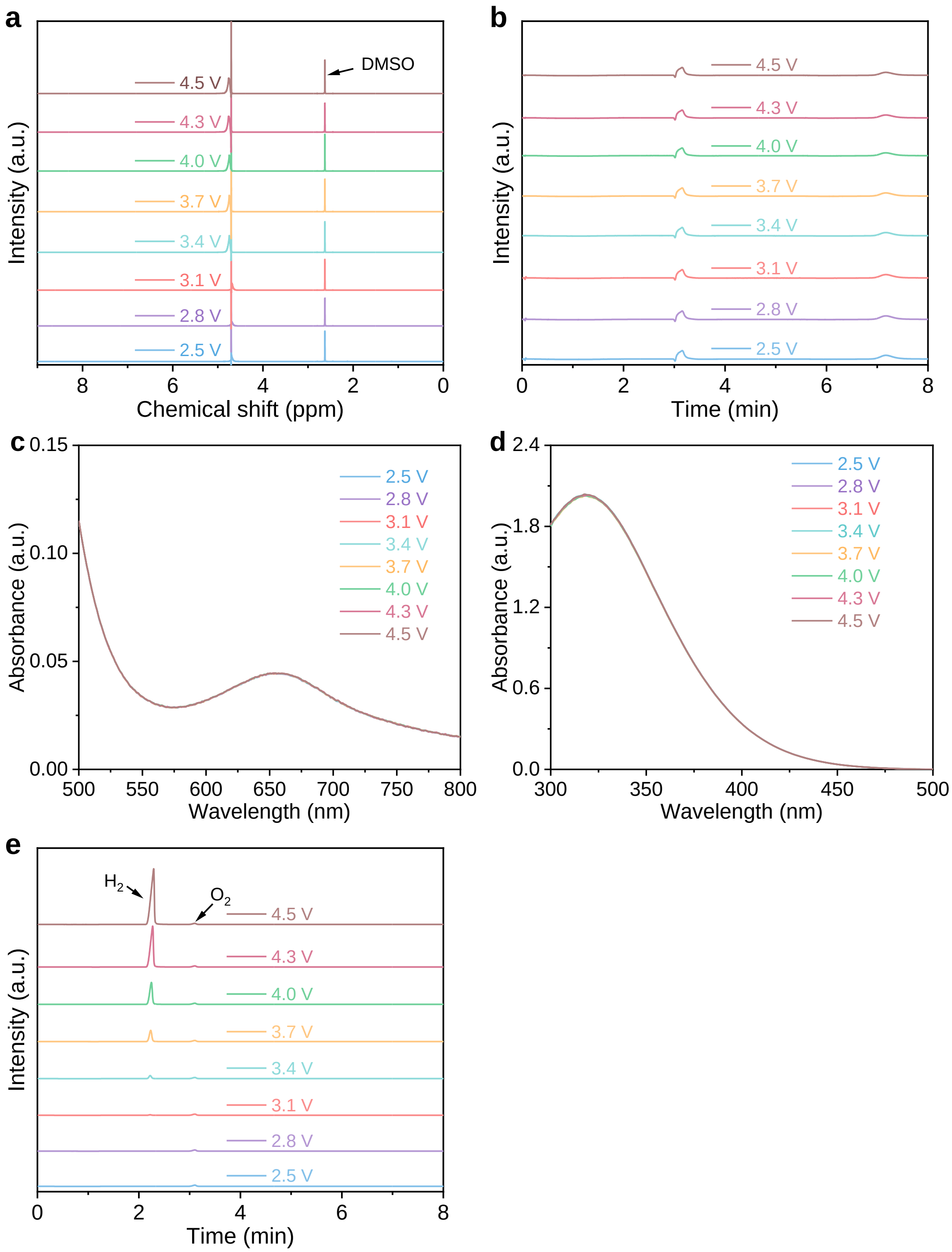

Supplementary Figure 33. ^{1}H NMR, GC and UV-vis measurements of products by GDL with flue gas as feedstock in flow cell with **CALF-20**-MMM. (a) ^{1}H NMR measurements of HCOOH, (b) GC profile of CO, (c) UV-vis measurements of NH_3, (d) UV-vis measurements of H_2O_2 and (e) GC profile of H_2 at the potentials of 2.5 V, 2.8 V, 3.1 V, 3.4 V, 3.7 V, 4.0 V, 4.3 V and 4.5 V, respectively.

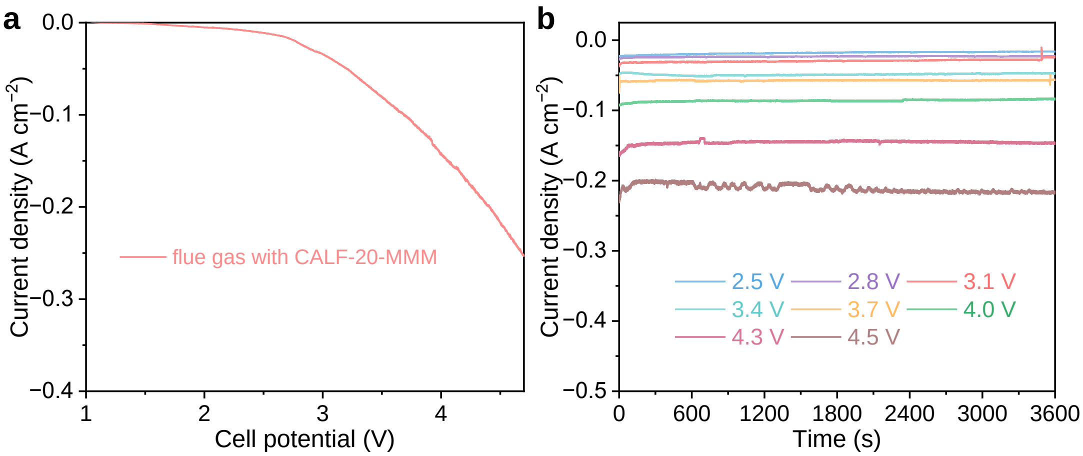

Supplementary Figure 34. Performance of eCO_2RR by GDL with flue gas as feedstock in flow cell with **CALF-20**-MMM. (a) LSV curve and (b) *i*-*t* curves.

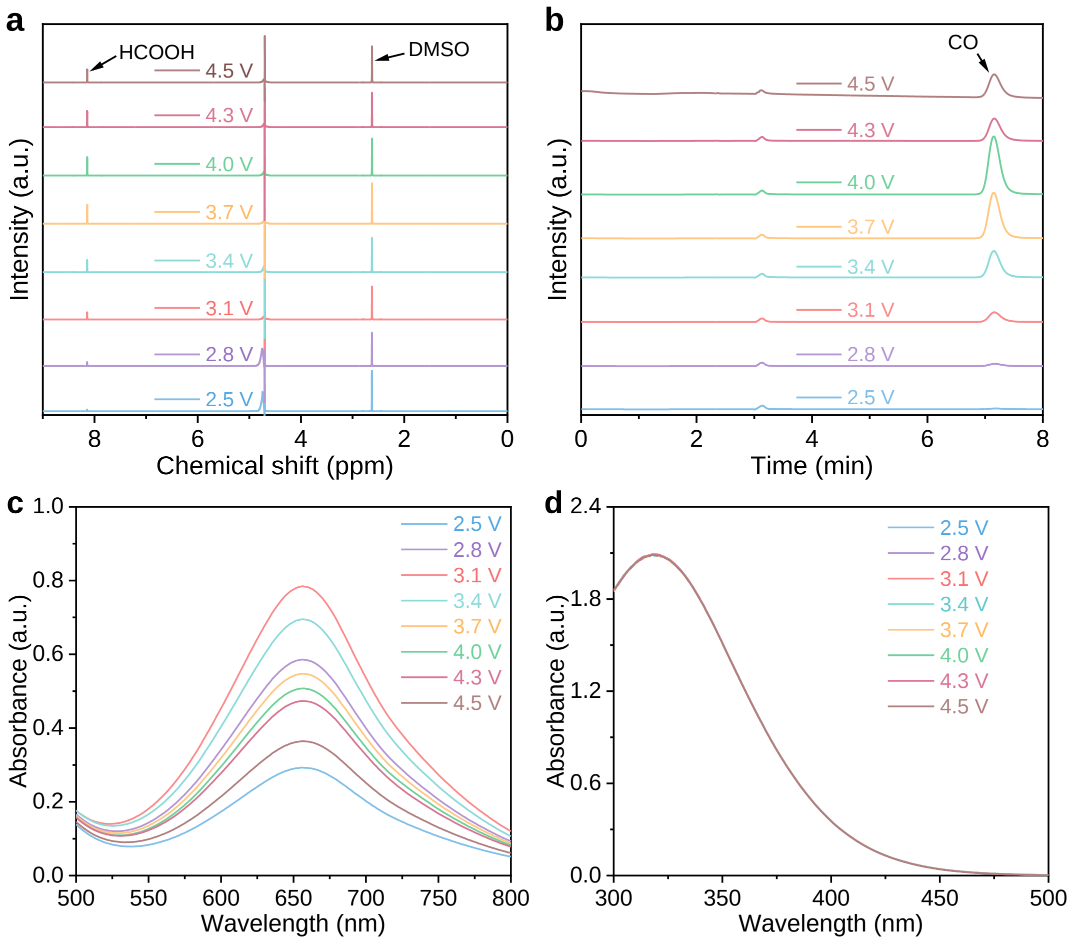

Supplementary Figure 35. ^{1}H NMR, GC and UV-vis measurements of products by Bi/GDL/**CALF-20** with flue gas as feedstock in flow cell. (a) ^{1}H NMR measurements of HCOOH, (b) GC profile of CO, (c) UV-vis measurements of NH_3 and (d) UV-vis measurements of H_2O_2 at the potentials of 2.5 V, 2.8 V, 3.1 V, 3.4 V, 3.7 V, 4.0 V, 4.3 V and 4.5 V, respectively.

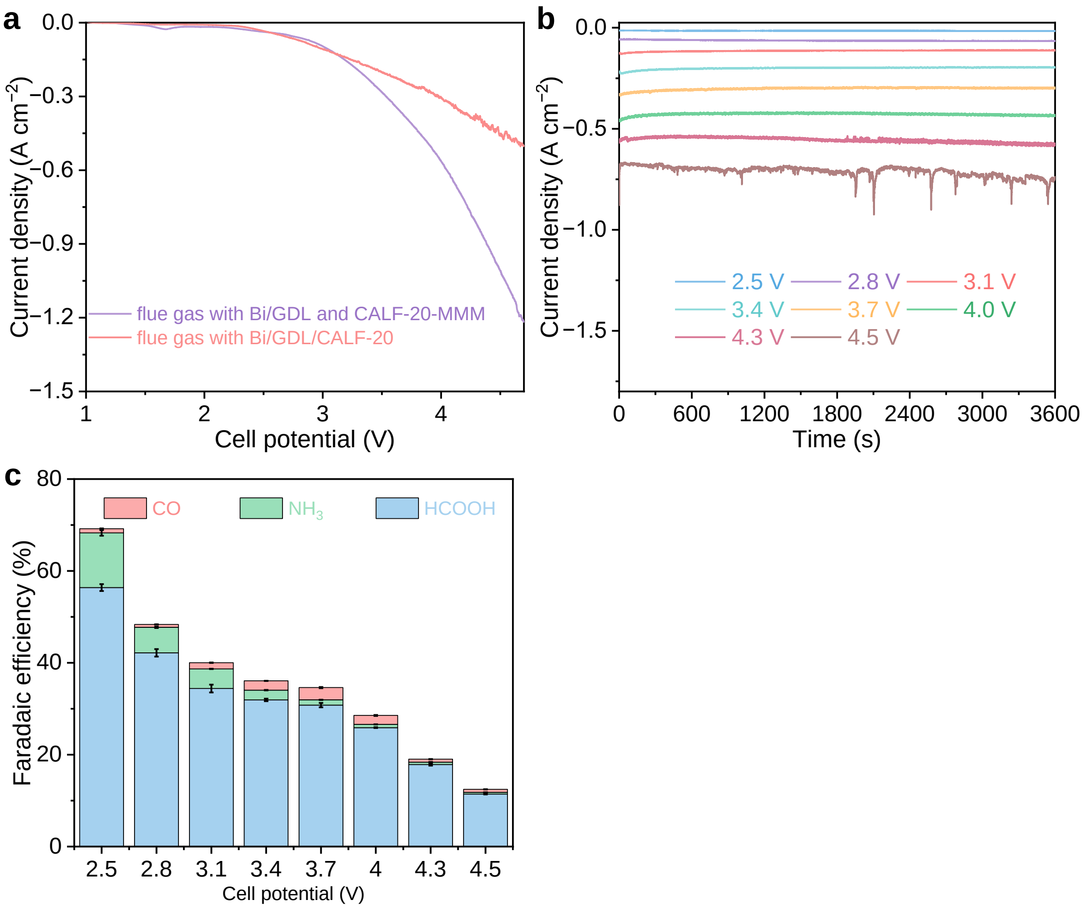

Supplementary Figure 36. Performance of eCO_2RR by Bi/GDL/**CALF-20** with flue gas as feedstock in flow cell. (a) LSV curve, (b) *i*-*t* curves and (c) FEs of HCOOH, CO and NH_3.

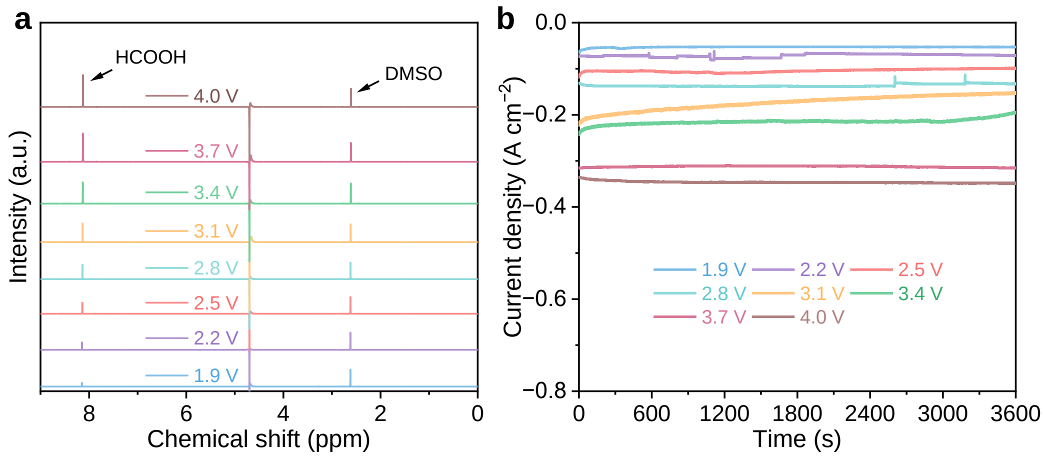

Supplementary Figure 37. ^{1}H NMR measurements and *i-t* curves by Bi/GDL with flue gas as feedstock in MEA-SSE electrolyzer with **CALF-20**-MMM. (a) ^{1}H NMR measurements and (b) *i-t* curves at the potentials of 1.9 V, 2.2 V, 2.5 V, 2.8 V, 3.1 V, 3.4 V, 3.7 V and 4.0 V, respectively.

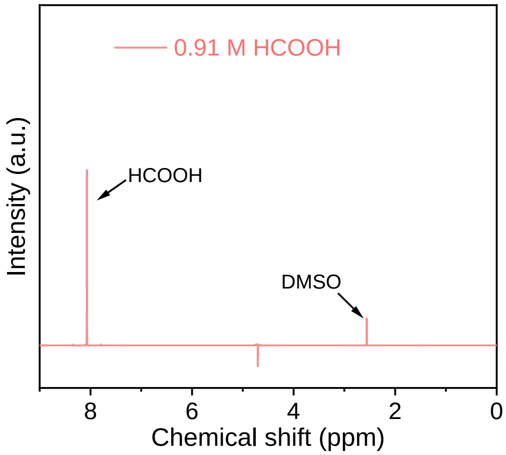

Supplementary Figure 38. ^{1}H NMR measurements after 300 h electrocatalysis. ^{1}H NMR measurements of HCOOH by Bi/GDL with flue gas as feedstock in MEA-SSE electrolyzer with **CALF-20**-MMM after 300 h electrocatalysis.

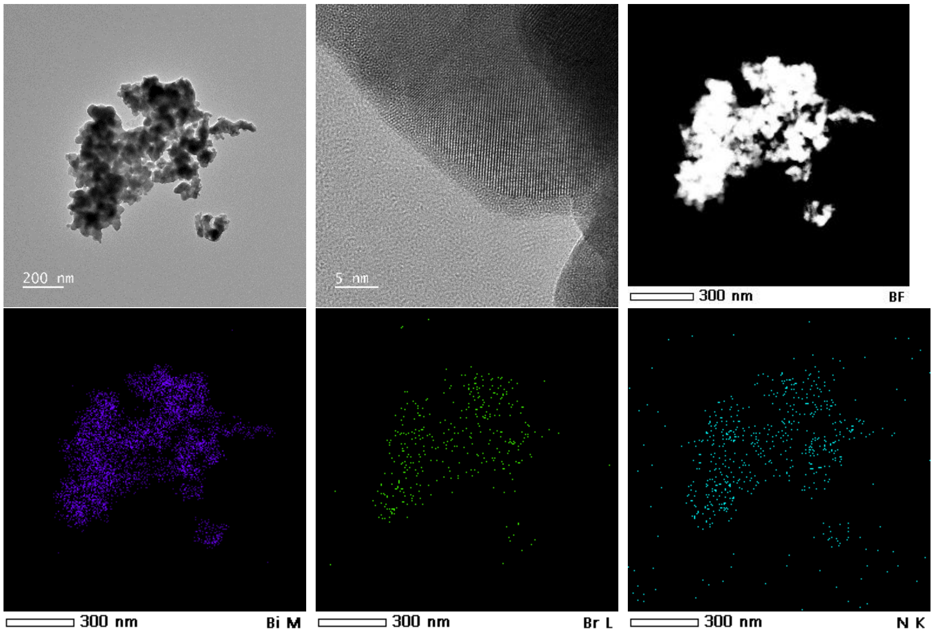

Supplementary Figure 39. HR-TEM image and EDS of Bi NPs after electrocatalysis.

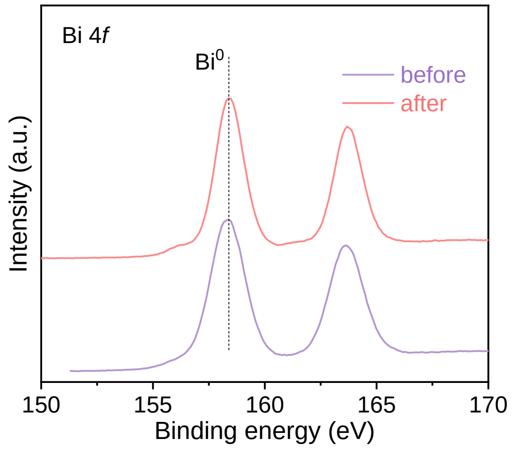

Supplementary Figure 40. XPS characterization of Bi NPs. The XPS of Bi 4*f* of Bi NPs. It can be seen that the valance of Bi is 0, indicating the structure stability of Bi NPs.

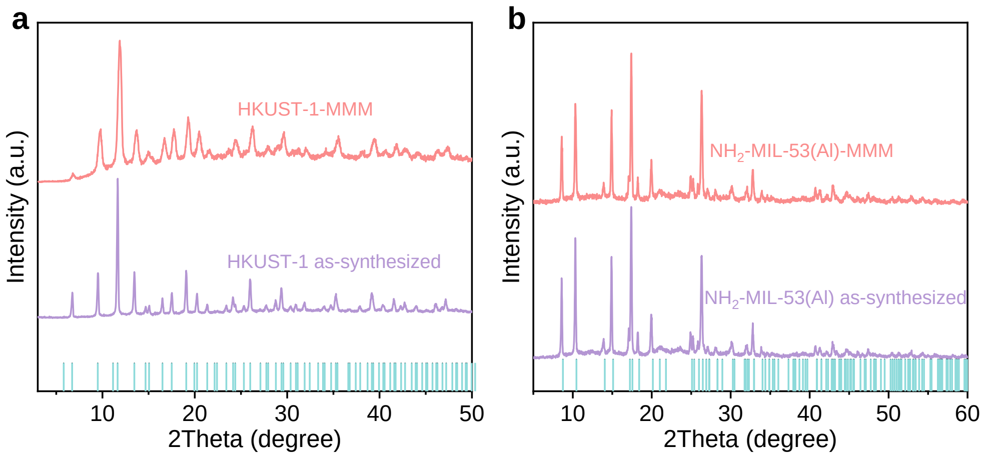

Supplementary Figure 41. PXRD characterization of **HKUST-1**, **NH_2-MIL-53(Al)**, and their MMMs. (a) PXRD patterns of **HKUST-1** and **HKUST-1**-MMM. (b) PXRD patterns of **NH_2-MIL-53(Al)** and **NH_2-MIL-53(Al)**-MMM.

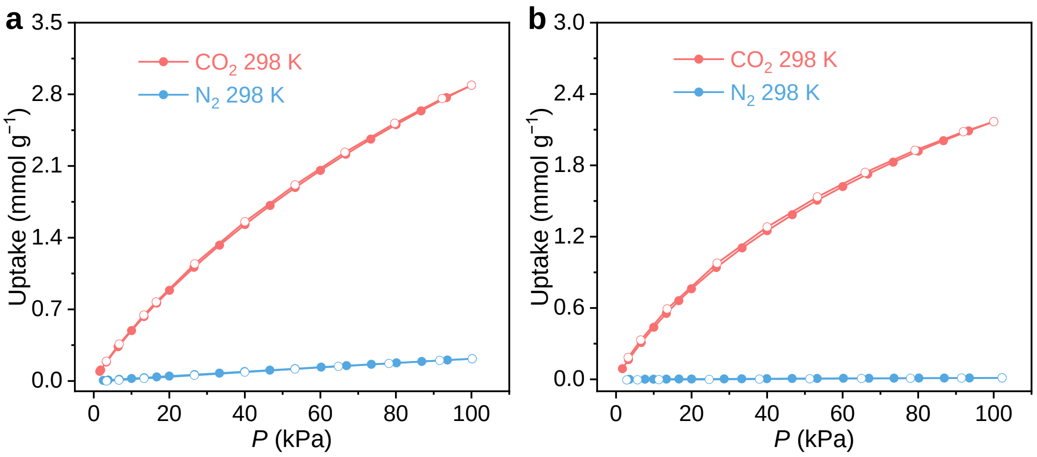

Supplementary Figure 42. CO_2 and N_2 sorption isotherms of **HKUST-1** and **NH_2-MIL-53(Al)** measured at 298 K. CO_2 and N_2 adsorption (solid) and desorption (open) isotherms of (a) **HKUST-1** and (b) **NH_2-MIL-53(Al)** measured at 298 K.

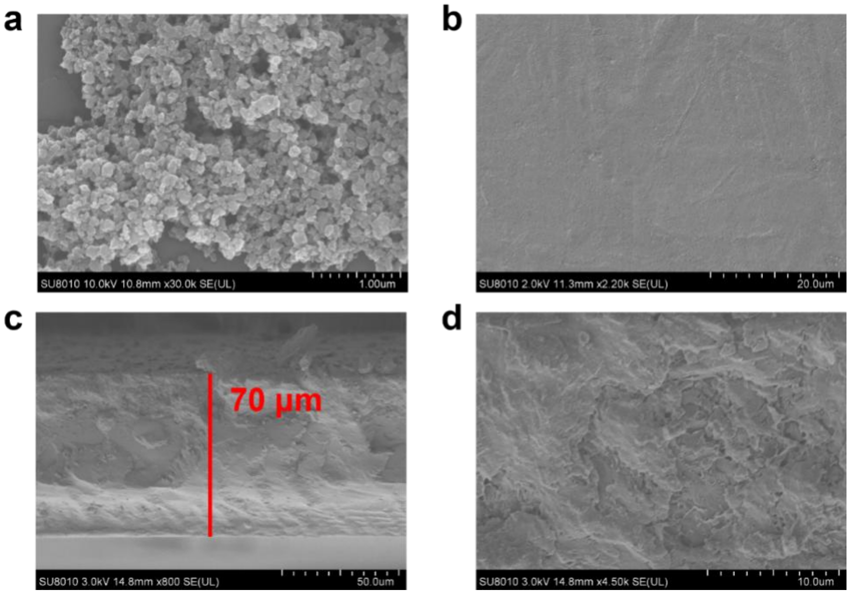

Supplementary Figure 43. SEM images of **HKUST-1** and **HKUST-1**-MMM. (a) SEM image of **HKUST-1**. (b) SEM image of the surface of **HKUST-1**-MMM. (c, d) SEM images of the cross section of **HKUST-1**-MMM at different resolutions.

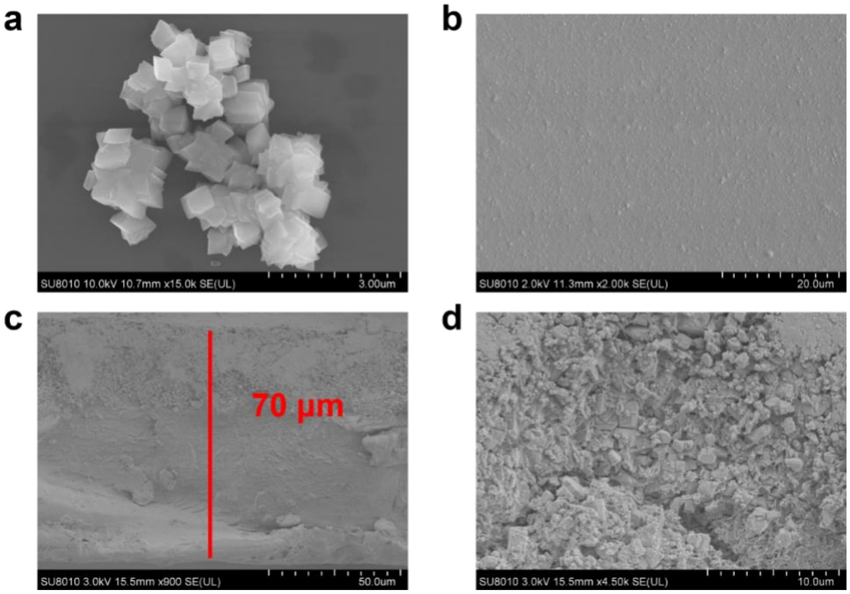

Supplementary Figure 44. SEM images of **NH_2-MIL-53(Al)** and **NH_2-MIL-53(Al)**-MMM. (a) SEM image of **NH_2-MIL-53(Al)**. (b) SEM image of the surface of **NH_2-MIL-53(Al)**-MMM. (c, d) SEM images of the cross section of **NH_2-MIL-53(Al)**-MMM at different resolutions.

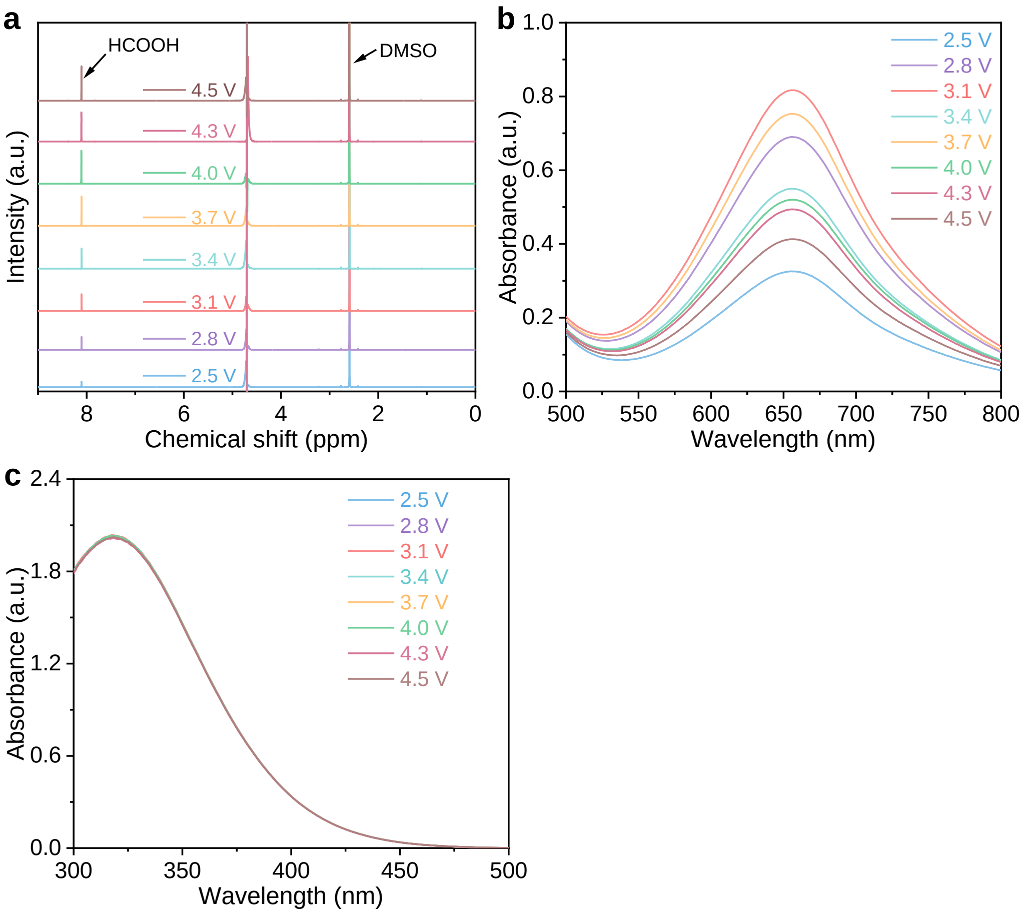

Supplementary Figure 45. ^{1}H NMR and UV-vis measurements of products by Bi/GDL with flue gas as feedstock in flow cell with **HKUST-1**-MMM. (a) ^{1}H NMR measurements of HCOOH and UV-vis measurements of (b) NH_3 and (c) H_2O_2 at the potentials of 2.5 V, 2.8 V, 3.1 V, 3.4 V, 3.7 V, 4.0 V, 4.3 V and 4.5 V, respectively.

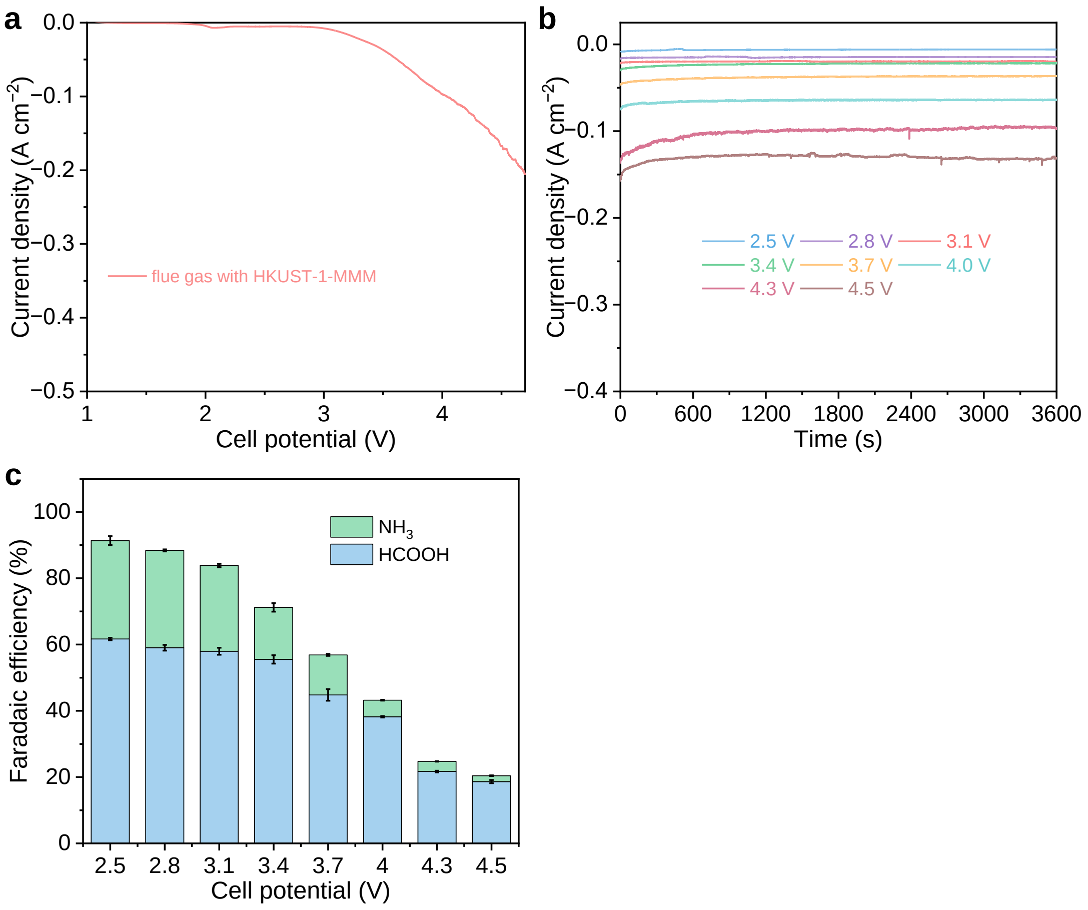

Supplementary Figure 46. Performance of eCO_2RR by Bi/GDL with flue gas as feedstock in flow cell with **HKUST-1**-MMM. (a) LSV curve, (b) *i*-*t* curves and (c) FEs of HCOOH and NH_3.

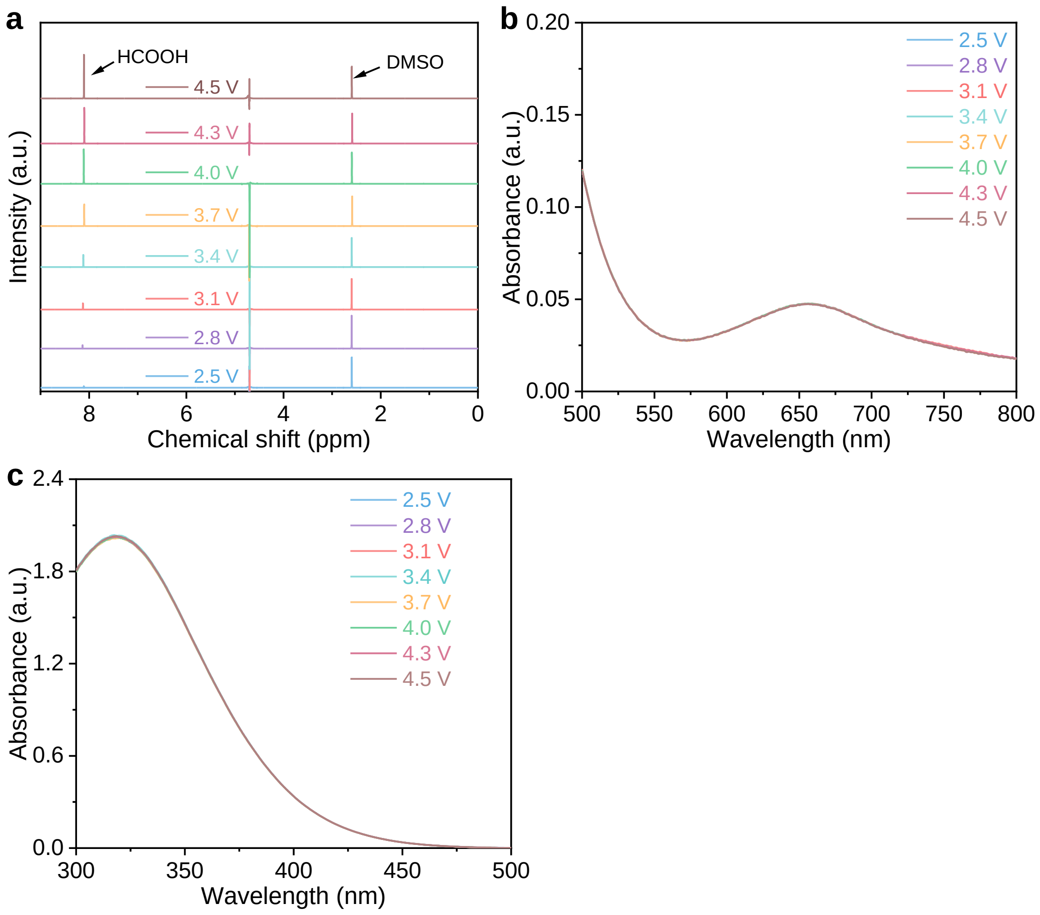

Supplementary Figure 47. ^{1}H NMR and UV-vis measurements of products by Bi/GDL with flue gas as feedstock in flow cell with **NH_2-MIL-53(Al)** MMM. (a) ^{1}H NMR measurements of HCOOH and UV-vis measurements of (b) NH_3 and (c) H_2O_2 at the potentials of 2.5 V, 2.8 V, 3.1 V, 3.4 V, 3.7 V, 4.0 V, 4.3 V and 4.5 V, respectively.

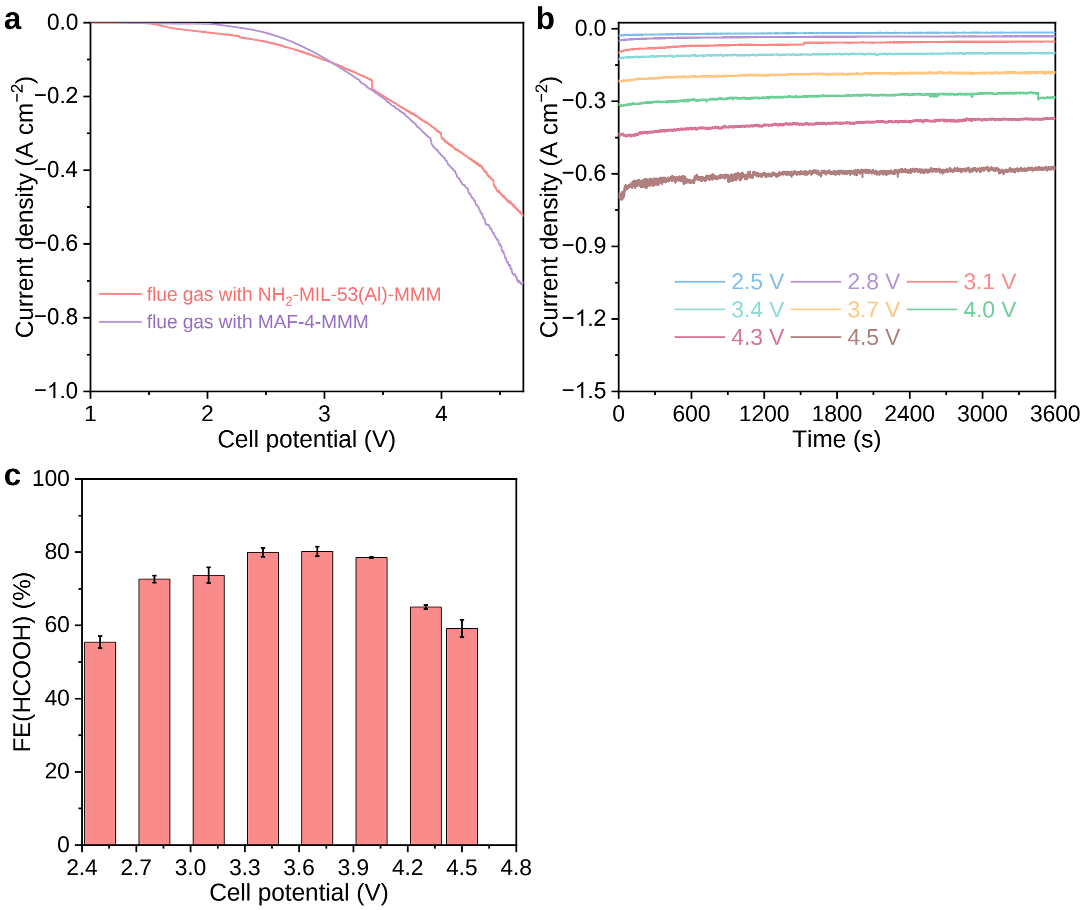

Supplementary Figure 48. Performance of eCO_2RR by Bi/GDL with flue gas as feedstock in flow cell with **NH_2-MIL-53(Al)**-MMM. (a) LSV curve, (b) *i-t* curves and (c) FE of HCOOH.

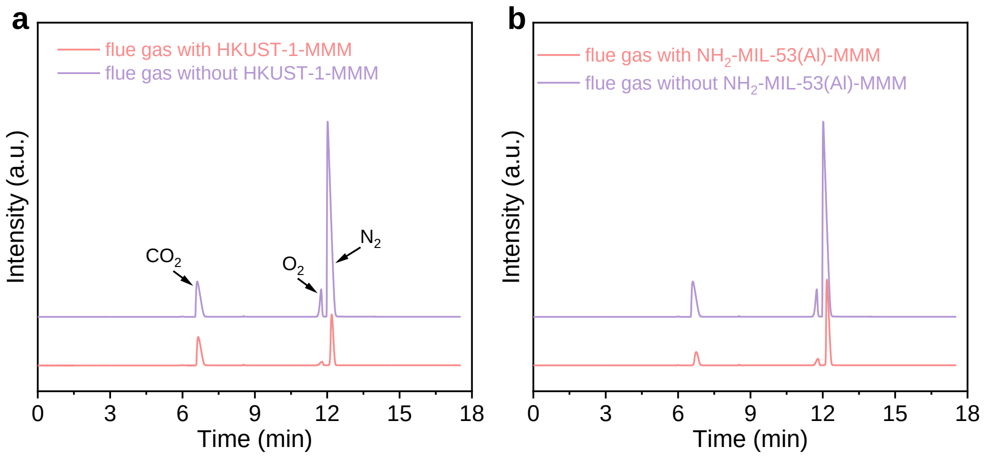

Supplementary Figure 49. GC profiles of CO_2. GC profiles of flue gas passing through (a) **HKUST-1**-MMM and (b) **NH_2-MIL-53(Al)**-MMM.

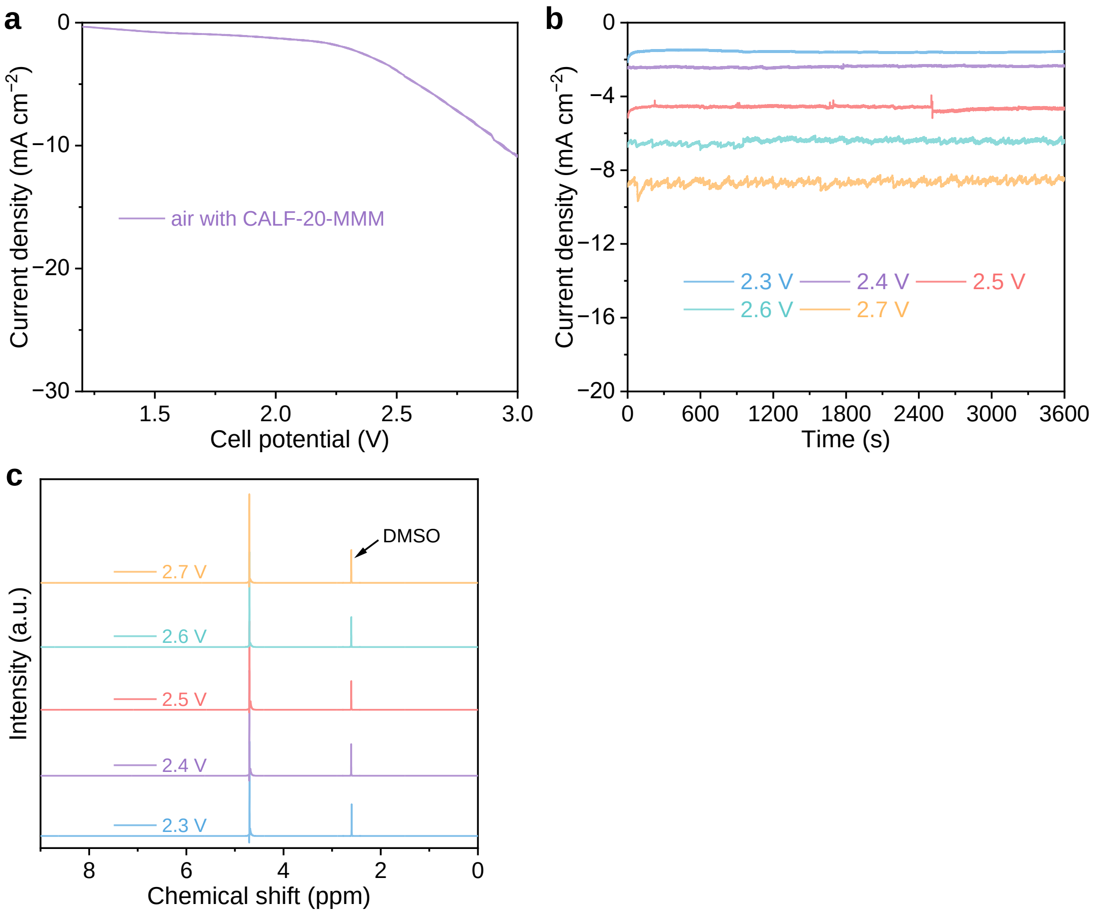

Supplementary Figure 50. Performance of eCO_2RR by Bi/GDL with air as feedstock in flow cell with **CALF-20**-MMM. (a) LSV curve, (b) *i*-*t* curves and (c) ^{1}H NMR measurement of HCOOH.

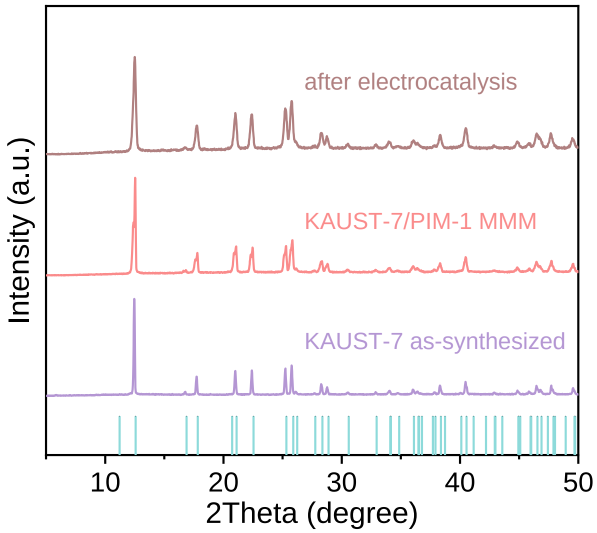

Supplementary Figure 51. PXRD characterization of **KAUST-7**. PXRD patterns of **KAUST-7** and **KAUST-7**-MMM.

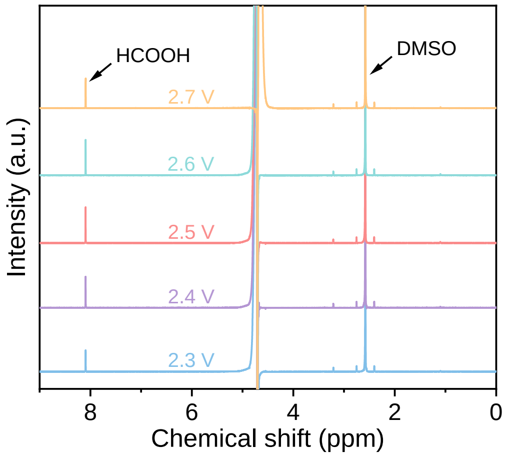

Supplementary Figure 52. ^{1}H NMR measurements of HCOOH by Bi/GDL with air as feedstock in flow cell with **KAUST-7**-MMM. ^{1}H NMR measurements of HCOOH at the potentials of 2.5 V, 2.8 V, 3.1 V, 3.4 V, 3.7 V, 4.0 V, 4.3 V and 4.5 V, respectively.

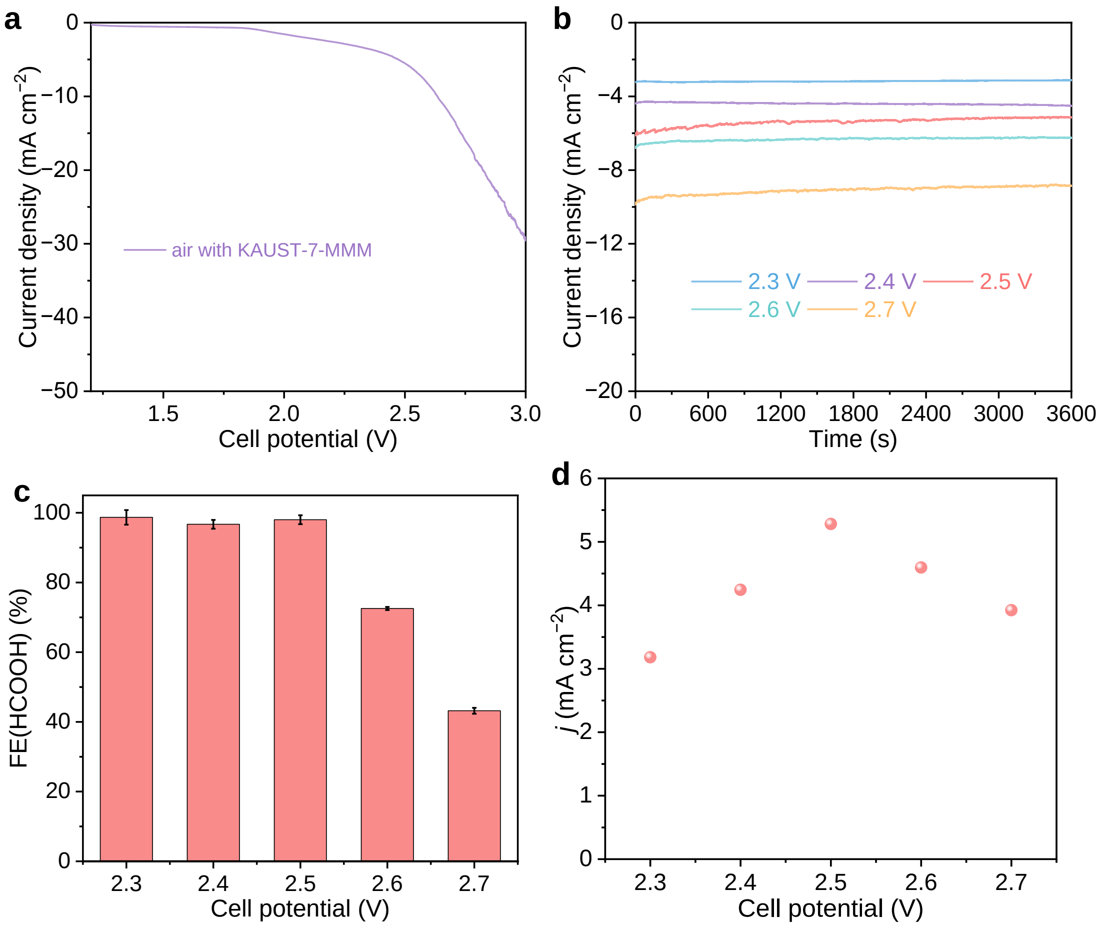

Supplementary Figure 53. Performance of eCO_2RR by Bi/GDL with air as feedstock in flow cell with **KAUST-7**-MMM. (a) LSV curve, (b) *i*-*t* curves, (c) FE of HCOOH and (d) partial current density of HCOOH.

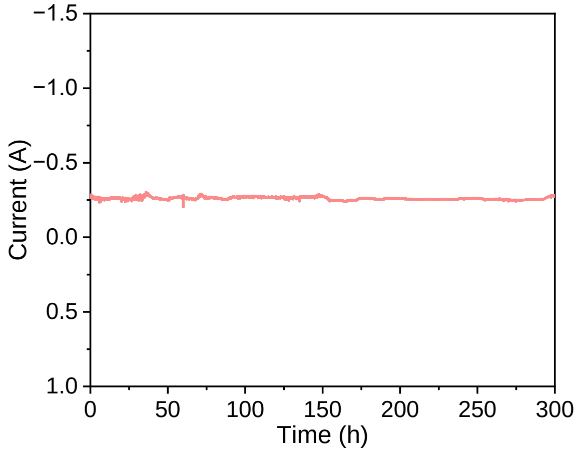

Supplementary Figure 54. Stability test. Stability test over 300 hours in electrocatalysis by Bi/GDL with air as feedstock in a gas-solid acidic membrane electrolyzer (window area of 10 × 10 cm^2) embedded with **KAUST-7**-MMM.

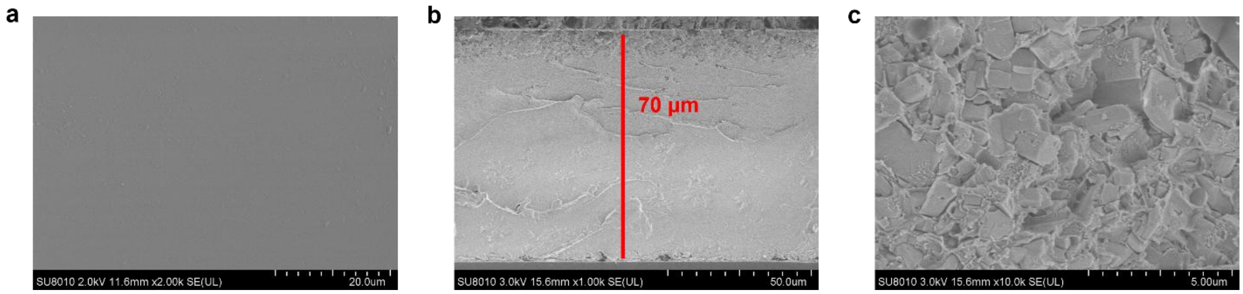

Supplementary Figure 55. SEM images of **KAUST-7**-MMM after electrocatalysis. (a) SEM image of the surface of **KAUST-7**-MMM after electrocatalysis. (b, c) SEM images of cross section of **KAUST-7**-MMM after electrocatalysis at different resolutions.

Catalyst	Electrolyte	FE (HCOOH)	Current density (mA cm^{-2})	Single-pass conversion efficiency for CO_2	Ref
**Bi NPs (flue gas)**	0.5 M K_2SO_4 + 0.05 M H_2SO_4	95.2%	820	79.3%	This work
		81.3%	1010	83.4%	
r-Pb	0.5 M K_2SO_4 + 0.05 M H_2SO_4	93%	600	91%	[4]
Bi RS	0.5 M K_2SO_4 + 0.05 M H_2SO_4	96.31%	471	/	[5]
Bi NS	3 M KCl + 0.05 M H_2SO_4	92.2%	237.1	27.4%	[6]
Cu_6Sn_5	3 M KCl +0.05 M H_2SO_4	96%	500	77.4%	[7]
BiS-1	0.5 M K_2SO_4 + 0.05 M H_2SO_4	93.2%	1212	65.3%	[8]

Supplementary Table 1. Comparison of eCO_2RR performances of different catalysts in flow cell.

Sample	Weight percentage of S element (%)
Pure CO_2	0.18
Flue gas	5.28
Flue gas with **MAF-4**- MMM	0.19
Flue gas with **CALF-20**-MMM	0.17
Flue gas with PIM-1 membrane	5.16
Flue gas with **HKUST-1**-MMM	4.63
Flue gas with **NH_2-MIL-53(Al)**-MMM	4.39

Supplementary Table 2. Element analysis of Bi NPs after electrocatalysis at different eCO_2RR conditions.

Catalyst	Electrolyte	Cell potential (V)	FE (HCOOH)	Current density (mA cm^{-2})	Stability (h)	Concentration of HCOOH (mol L^{-1})	Ref
**Bi NPs (flue gas)**	H_2	4.0	94%	340	300	0.91	This work
Bi-HHTP (Dilute CO_2 15 vol.%)	H_2	2.7	90%	85	30	0.2	[9]
2D-Bi	0.5 M H_2SO_4	3	80%	30	100	0.1	[10]
Cu_6Sn_5	0.5 M H_2SO_4	3.7	96%	100	130	0.36	[7]
Pb_1Cu	0.5 M H_2SO_4	3.45	85%	100	180	0.1	[11]
RD-Bi	0.5 M H_2SO_4	4.1	80%	200	300	2	[12]
Bi_2S_3-derived	0.5 M H_2SO_4	4.2	93%	275	280	3.5	[13]
In_2O_3@C	1 M H_2SO_4	3.6	81%	30	3	0.12	[14]

Supplementary Table 3. Comparison of eCO_2RR performances of different catalysts in MEA-SSE electrolyzer.

Catalyst	Electrolyte	FE (%)	Flow rate of CO_2 (sccm)	Conversion efficiency (%)	Stability (h)	Yield rate (μmol h^{-1} g^{-1})	Ref
**Bi NPs**	0.5 M K_2SO_4 + 0.05 M H_2SO_4	95	200	50.3	300	177200	This work
Cu-PCN@PVDF	NA	96.24	NA	NA	10.5	33	*[15]*
nano-Cu	1 M KOH	NA	0.5	79	3.5	NA	[16]

Supplementary Table 4. Comparison of eCO_2RR performances of different catalysts with air as feedstock in flow cell.

References

1. Wang Q-H, Yu L-J, Liu Y *et al.* Methods for the detection and determination of nitrite and nitrate: A review. *Talanta* 2017; **165**: 709-20.
2. Zhu D, Zhang L, Ruther RE *et al.* Photo-illuminated diamond as a solid-state source of solvated electrons in water for nitrogen reduction. *Nat Mater* 2013; **12**: 836-41.
3. Chen Y, Zhen C, Chen Y *et al.* Oxygen functional groups regulate cobalt-porphyrin molecular electrocatalyst for acidic H_2O_2 electrosynthesis at industrial-level current. *Angew Chem Int Ed* 2024; **63**: e202407163.
4. Fang W, Guo W, Lu R *et al.* Durable CO_2 conversion in the proton-exchange membrane system. *Nature* 2024; **626**: 86-91.
5. Chi LP, Niu ZZ, Zhang YC *et al.* Efficient and stable acidic CO_2 electrolysis to formic acid by a reservoir structure design. *Proc Natl Acad Sci U S A* 2023; **120**: e2312876120.
6. Qiao Y, Lai W, Huang K *et al.* Engineering the Local Microenvironment over Bi Nanosheets for Highly Selective Electrocatalytic Conversion of CO_2 to HCOOH in Strong Acid. *ACS Catal* 2022; **12**: 2357-64.
7. Yu X, Xu Y, Li L *et al.* Coverage enhancement accelerates acidic CO_2 electrolysis at ampere-level current with high energy and carbon efficiencies. *Nat Commun* 2024; **15**: 1711.
8. Angew Chem Int Ed EnglJiang Z, Ren S, Cao X *et al.* pH-Universal electrocatalytic CO_2 reduction with ampere-level current density on doping-engineered bismuth sulfide. *Angew Chem Int Ed* 2024; **63**: e202408412.
9. Zhao ZH, Huang JR, Huang DS *et al.* Efficient capture and electroreduction of dilute CO_2 into highly pure and concentrated formic acid aqueous solution. *J Am Chem Soc* 2024; **146**: 14349-56.
10. Xia C, Zhu P, Jiang Q *et al.* Continuous production of pure liquid fuel solutions via electrocatalytic CO_2 reduction using solid-electrolyte devices. *Nat Energy* 2019; **4**: 776-85.
11. Zheng T, Liu C, Guo C *et al.* Copper-catalysed exclusive CO_2 to pure formic acid conversion via single-atom alloying. *Nat Nanotechnol* 2021; **16**: 1386-93.
12. Zhang C, Hao X, Wang J *et al.* Concentrated formic acid from CO_2 electrolysis for directly driving fuel cell. *Angew Chem Int Ed* 2024; **63**: e202317628.
13. Lin L, He X, Zhang XG *et al.* A nanocomposite of bismuth clusters and $Bi_2O_2CO_3$ sheets for highly efficient electrocatalytic reduction of CO_2 to formate. *Angew Chem Int Ed* 2023; **62**: e202214959.
14. Wang Z, Zhou Y, Liu D *et al.* Carbon-Confined Indium Oxides for Efficient Carbon Dioxide Reduction in a Solid-State Electrolyte Flow Cell. *Angew Chem Int Ed* 2022; **61**: e202200552.
15. Wang N, Jiang W, Yang J *et al.* Contact-electro-catalytic CO_2 reduction from ambient air. *Nat Commun* 2024; **15**: 5913.
16. Chen Y, Kan M, Yan S *et al.* Electroreduction of air-level CO_2 with high conversion efficiency. *Chin J Catal* 2022; **43**: 1703-09.
